# Metal and Ligand Effects on the Construction of Divalent Coordination Polymers Based on bis-Pyridyl-bis-amide and Polycarboxylate Ligands

**DOI:** 10.3390/polym9120691

**Published:** 2017-12-08

**Authors:** Miao-Ning Chang, Xiang-Kai Yang, Pradhumna Mahat Chhetri, Jhy-Der Chen

**Affiliations:** Department of Chemistry, Chung-Yuan Christian University, Chung-Li 32023, Taiwan; applemiao531@gmail.com (M.-N.C.); xiangkaishulin@gmail.com (X.-K.Y.); mahatp@gmail.com (P.M.C.)

**Keywords:** coordination polymer, bis-pyridyl-bis-amide, polycarboxylate, photodegradation

## Abstract

Ten coordination polymers constructed from divalent metal salts, polycarboxylic acids, and bis-pyridyl-bis-amide ligands with different donor atom positions and flexibility are reported. They were structurally characterized by single-crystal X-ray diffraction. The ten coordination polymers are as follows: (**1**) {[Ni(**L^1^**)(3,5-PDA)(H_2_O)_3_]·2H_2_O}*_n_* (**L^1^** = *N*,*N*′-di(3-pyridyl)suberoamide, 3,5-H_2_PDA = 3,5-pyridinedicarboxylic acid); (**2**) {[Ni_2_(**L^1^**)_2_(1,3,5-HBTC)_2_(H_2_O)_4_]·H_2_O}*_n_* (1,3,5-H_3_BTC = 1,3,5-benzenetricarboxylic acid); (**3**) {[Ni(**L^2^**)(5-*tert*-IPA)(H_2_O)_2_]·2H_2_O}*_n_* (**L^2^** = *N*,*N*′-di(3-pyridyl)adipoamide, 5-*tert*-H_2_IPA = 5-*tert*-butylisophthalic acid); (**4**) [Ni(**L^3^**)_1.5_(5-*tert*-IPA)]*_n_* (**L^3^** = *N*,*N*′-di(4-pyridyl)adipoamide); (**5**) [Co(**L^1^**)(1,3,5-HBTC)(H_2_O)]*_n_*; (**6**) {[Co_3_(**L^1^**)_3_(1,3,5-BTC)_2_(H_2_O)_2_]·6H_2_O}*_n_*; (**7**) [Cu(**L^4^**)(AIPA)]*_n_* (**L^4^** = *N*,*N*′-bis(3-pyridinyl)terephthalamide, H_2_AIPA = 5-acetamido isophthalic acid); (**8**) {[Cu(**L^2^**)_0.5_(AIPA)]·MeOH}*_n_*; (**9**) {[Zn(**L^4^**)(AIPA)]·2H_2_O}*_n_*; and (**10**) {[Zn(**L^2^**)(AIPA)]·2H_2_O}*_n_*. Complex **1** forms a 1D chain and **2** is a two-fold interpenetrated 2D layer with the **sql** topology, while **3** is a 2D layer with the **hcp** topology and **4** shows a self-catenated 3D framework with the rare (4^2^·6^7^·8)-**hxg**-d-5-C2/c topology. Different Co/1,3,5-H_3_BTC ratios were used to prepare **5** and **6**, affording a 2D layer with the **sql** topology and a 2D layer with the (4·8^5^)_2_(4)_2_(8^3^)_2_(8) topology that can be further simplified to an **hcp** topology. While complex **7** is a 2D layer with the (4^2^·6^7^·8)(4^2^·6)-3,5L2 topology and **8** is a 2-fold interpenetrated 3D framework with the **pcu** topology, complexes **9** and **10** are self-catenated 3D frameworks with the (4^24^·6^4^)-8T2 and the (4^4^·6^10^·8)-**mab** topologies, respectively. The effects of the identity of the metal center, the ligand isomerism, and the flexibility of the spacer ligands on the structural diversity of these divalent coordination polymers are discussed. The luminescent properties of **9** and **10** and their photocatalytic effects on the degradation of dyes are also investigated.

## 1. Introduction

Owing to the interesting structures of coordination polymers (CPs) and their potential applications in magnetism, luminescence, catalysis, gas storage, and sensing, CPs have been extensively studied and discussed by scientists during recent years [1,2,3,4]. Through the self-assembly process, the coordination of spacer ligands to the metal ions may lead to the formation of infinite one-dimensional (1D), two-dimensional (2D), or three-dimensional (3D) CPs. Judicial choice of the metal-ion template and spacer ligands with diverse functionalities and flexibility may form well-defined frameworks.

The bis-pyridyl-bis-amide (bpba) ligands are intriguing ligands that can be tailored to prepare diverse CPs [5]. Most of the bpba ligands are flexible, although some are semi-rigid. While diverse bpba-based CPs have been reported, the control of structural dimensionality remains a challenge and the governing factors are less ascertained [5,6]. We have shown that reactions of the flexible *N*,*N*′-di(3-pyridyl)suberoamide (**L^1^**) with Cu(II) salts in the presence of the isomeric phenylenediacetic acids under hydrothermal conditions afforded a 3D CP with the (4^2^·6^5^·8^3^)(4^2^·6)-3,5T1 topology, a 5-fold interpenetrated 3D CP with the (6^5^·8)-**cds** topology, and the first 1D self-catenated CP [7]. We have recently further shown that by manipulating the isomeric effect of the dicarboxylate ligands, self-catenated CPs incorporating the flexible bpba ligands can be expected [8].

**Figure 1:**
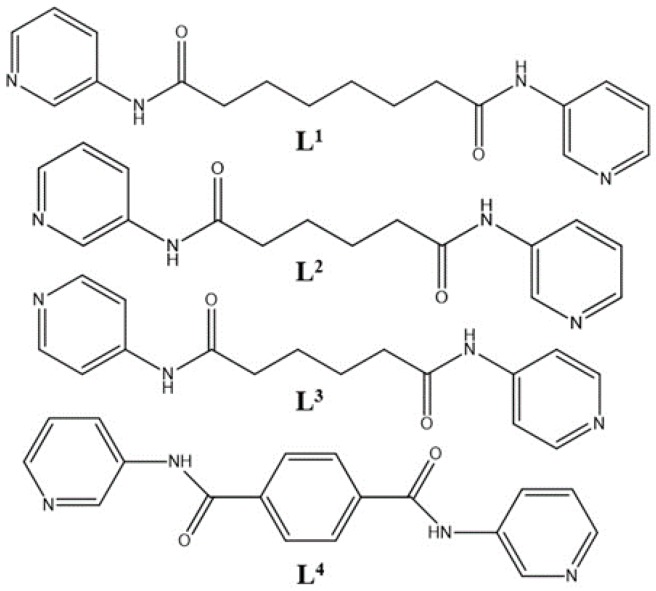
Structures of **L^1^**, **L^2^**, **L^3^**, and **L^4^**.

**Figure 2:**
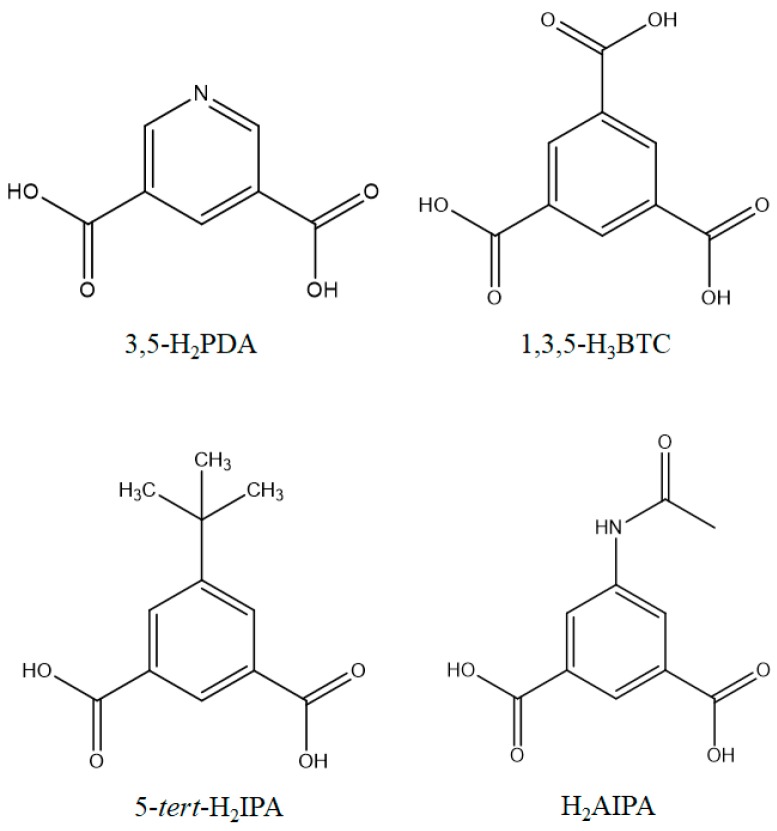
Structures of the polycarboxylic acids.

To investigate the structure-directing roles of the spacer ligands on the construction of bpba-based CPs by changing their donor atom positions and flexibility, the flexible **L^1^**, *N*,*N*′-di(3-pyridyl)adipoamide (**L^2^**), and *N*,*N*′-di(4-pyridyl)adipoamide (**L^3^**), and the semi-rigid *N*,*N*′-bis(3-pyridinyl)terephthalamide (**L^4^**) (Figure 1) were used to react with different divalent metal salts and auxiliary polycarboxylic acids (Figure 2). Various CPs with fascinating topology and interesting properties were prepared. Herein, we report the synthesis and crystal structures of the following: (**1**) {[Ni(**L^1^**)(3,5-PDA)(H_2_O)_3_]·2H_2_O}*_n_* (**L^1^** = *N*,*N*′-di(3-pyridyl)suberoamide, 3,5-H_2_PDA = 3,5-pyridinedicarboxylic acid); (**2**) {[Ni_2_(**L^1^**)_2_(1,3,5-HBTC)_2_(H_2_O)_4_]·H_2_O}*_n_* (1,3,5-H_3_BTC = 1,3,5-benzenetricarboxylic acid); (**3**) {[Ni(**L^2^**)(5-*tert*-IPA)(H_2_O)_2_]·2H_2_O}*_n_* (**L^2^** = *N*,*N*′-di(3-pyridyl)adipoamide, 5-*tert*-H_2_IPA = 5-*tert*-butylisophthalic acid); (**4**) [Ni(**L^3^**)_1.5_(5-*tert*-IPA)]*_n_* (**L^3^** = *N*,*N*′-di(4-pyridyl)adipoamide); (**5**) [Co(**L^1^**)(1,3,5-HBTC)(H_2_O)]*_n_*; (**6**) {[Co_3_(**L^1^**)_3_(1,3,5-BTC)_2_(H_2_O)_2_]·6H_2_O}*_n_*; (**7**) [Cu(**L^4^**)(AIPA)]*_n_* (**L^4^** = *N*,*N*′-bis(3-pyridinyl)terephthalamide, H_2_AIPA = 5-acetamido isophthalic acid), (**8**) {[Cu(**L^2^**)_0.5_(AIPA)]·MeOH}*_n_*; (**9**) {[Zn(**L^4^**)(AIPA)]·2H_2_O}*_n_*; and (**10**) {[Zn(**L^2^**)(AIPA)]·2H_2_O}*_n_*. The luminescent and catalytic properties of some applicable complexes were also investigated.

## 2. Experimental Section

### 2.1. Materials

The reagent Ni(OAc)_2_·4H_2_O, 3,5-pyridinedicarboxylic acid, and 1,3,5-benzenetricarboxylic acid were purchased from Alfa Aesar Co. (Heysham, UK); Cu(OAc)_2_·H_2_O and Zn(OAc)_2_·2H_2_O from SHOWA Co. (Tokyo, Japan); Co(OAc)_2_·4H_2_O from J. T. Baker Co. (Phillipsburg, MO, USA); and 5-*tert*-butylisophthalic acid from ALDRICH Co. (St. Louis, MO, USA) *N*,*N*′-di(3-pyridyl)suberoamide (**L^1^**), *N*,*N*′-di(3-pyridyl)adipoamide (**L^2^**), *N*,*N*′-di(4-pyridyl)adipoamide (**L^3^**), *N*,*N*′-bis(3-pyridinyl)terephthalamide (**L^4^**), and 5-acetamido isophthalic acid (H_2_AIPA) were prepared according to published procedures [5,9].

### 2.2. Instruments

IR spectra (KBr disk) were performed on a JASCO FT/IR-460 plus spectrometer (JASCO, 28600 Mary’s Court City, USA). Elemental analyses were carried out on a PE 2400 series II CHNS/O analyzer (PerkinElmer, Boston, MA, USA) or an elementary VarioEL-III analyzer (Elementar Americas Inc., New Jersey, NJ, USA). Emission spectra were obtained using a Hitachi F-4500 spectrometer (Hitachi, Tokyo, Japan). Powder X-ray diffraction measurements were conducted on a Bruker D_2_ PHASER diffractometer (Bruker, Billerica, MA, USA) with CuK_α_ (λ_α_ = 1.54 Å) radiation. The solution UV–Vis absorption spectra were recorded using an UV-2450 spectrophotometer (Shimadzu, Kyoto, Japan).

### 2.3. Preparations

#### 2.3.1. General Procedure

A mixture of metal salts, bpba, and polycarboxylic acids in an appropriate volume of solvent was sealed in a 23 mL Teflon-lined stainless steel autoclave, which was heated under autogenous pressure to 100 °C for two days. The reaction system was then cooled to room temperature at a rate of 2 °C per hour. Crystals suitable for single-crystal X-ray diffraction were collected and washed with methanol and water.

#### 2.3.2. {[Ni(**L^1^**)(3,5-PDA)(H_2_O)_3_]·2H_2_O}*_n_*, c**1**

A mixture of Ni(OAc)_2_·4H_2_O (0.025 g, 0.10 mmol), **L^1^** (0.033 g, 0.10 mmol), and 3,5-H_2_PDA (0.017 g, 0.10 mmol) in 10 mL of NaOH (0.01 M) solution was used. Yield: 0.028 g (44%). Anal. Calcd for C_25_H_35_NiN_5_O_11_ (*M*_W_ = 640.29): C, 46.93; H, 5.51; N, 10.95%. Found: C, 46.42; H, 5.24; N, 10.73%. FT–IR (cm^−1^): 3414(s), 3253(s), 3084(m), 2905(m), 2360(w), 1672(m), 1608(s), 1550(s), 1479(w), 1428(s), 1376(s), 1340(w), 1320(w), 1274(m), 1236(w), 1175(w), 1057(w), 967(w), 812(m), 700(m), 641(w), 579(w).

#### 2.3.3. {[Ni_2_(**L^1^**)_2_(1,3,5-HBTC)_2_(H_2_O)_4_]·H_2_O}*_n_*, c**2**

A mixture of Ni(OAc)_2_·4H_2_O (0.025 g, 0.10 mmol), **L^1^** (0.033 g, 0.10 mmol), and 1,3,5-H_3_BTC (0.021 g, 0.10 mmol) in 5 mL of NaOH (0.01 M) solution was used. Yield: 0.046 g (36%). Anal. C_54_H_64_Ni_2_N_8_O_22_ (*M*_W_ = 1294.54) (**2** + 1 H_2_O): C, 50.10; H, 4.98; N, 8.66%. Found: C, 49.78; H, 5.02; N, 8.65%. FT–IR (cm^−1^): 3414(s), 3253(s), 3084(m), 2905(m), 2360(w), 1672(m), 1608(s), 1550(s), 1479(w), 1428(s), 1376(s), 1340(w), 1320(w), 1274(m), 1236(w), 1175(w), 1057(w), 967(w), 812(m), 700(m), 641(w), 579(w).

#### 2.3.4. {[Ni(**L^2^**)(5-*tert*-IPA)(H_2_O)_2_]·2H_2_O}*_n_*, c**3**

A mixture of Ni(OAc)_2_·4H_2_O (0.025 g, 0.10 mmol), **L^2^** (0.030 g, 0.10 mmol) and 5-*tert*-H_2_IPA (0.022 g, 0.10 mmol) in 10 mL H_2_O was used. Yield: 0.0423 g (65.28%). Anal. Calcd for C_28_H_38_NiN_4_O_10_ (*M*_W_ = 649.31): C, 51.84; H, 5.90; N, 8.64%. Found: C, 51.56; H, 5.92; N, 8.59%. FT–IR (cm^−1^): 3251(w), 3069(w), 2960(w), 1686(m), 1614(m), 1553(s), 1489(s), 1434(m), 1408(m), 1372(s), 1294(m), 1123(w), 1060(w), 1030(w).

#### 2.3.5. [Ni(**L^3^**)_1.5_(5-*tert*-IPA)]*_n_*, c**4**

A mixture of Ni(OAc)_2_·4H_2_O (0.025 g, 0.10 mmol), **L^3^** (0.030 g, 0.10 mmol), and 5-*tert*-H_2_IPA (0.022 g, 0.10 mmol) in 10 mL of NaOH (0.01 M) solution was used. Yield: 0.0389 g (53.58%). Anal. Calcd for C_36_H41NiN_6_O_8_ (*M*_W_ = 744.44) (**4** + 1 H_2_O): C, 58.08; H, 5.55; N, 11.29%. Found: C, 58.00; H, 5.47; N, 11.16%. FT–IR (cm^−1^): 3614(w), 3521(w), 3443(w), 3267(w), 3077(w), 2959(w), 1712(m), 1600(s), 1567(m), 1514(s), 1424(m), 1354(m), 1331(m), 1299(m), 1204(m), 1152(w), 1133(m), 1063(w), 1021(w).

#### 2.3.6. [Co(**L^1^**)(1,3,5-HBTC)(H_2_O)]*_n_*, c**5**

A mixture of Co(OAc)_2_·4H_2_O (0.025 g, 0.10 mmol), **L^1^** (0.033 g, 0.10 mmol), and 1,3,5-H_3_BTC (0.021 g, 0.10 mmol) in 10 mL of NaOH (0.01 M) solution was used. Yield: 0.046 g (36%). Anal. Calcd for C_27_H_28_CoN_4_O_9_ (*M*_W_ = 611.46): C, 53.02; H, 4.62; N, 9.16%. Found: C, 52.60; H, 4.73; N, 9.18%. FT–IR (cm^−1^): 3850(w), 3835(w), 3289(m), 2934(m), 2864(m), 1868(w), 1679(s), 1613(s), 1552(s), 1487(s), 1427(m), 1371(s), 1323(m), 1294(m), 1234(s), 1188(s), 1103(m), 1052(w), 995(w), 937(w), 913(w), 804(m), 679(m), 642(m), 557(w), 518(w), 490(w), 446(w), 423(w), 116(w).

#### 2.3.7. {[Co_3_(**L^1^**)_3_(1,3,5-BTC)_2_(H_2_O)_2_]·6H_2_O}*_n_*, c**6**

A mixture of Co(OAc)_2_·4H_2_O (0.025 g, 0.10 mmol), **L^1^** (0.033 g, 0.10 mmol), and 1,3,5-H_3_BTC (0.015 g, 0.05 mmol) in 10 mL of NaOH (0.01 M) solution was used. Yield: 0.046 g (36%). Anal. Calcd for C_72_H_88_Co_3_N_12_O_26_ (*M*_W_ = 1714.33): C, 50.43; H, 5.18; N, 9.81%. C_72_H_84_Co_3_N_12_O_24_ (*M*_W_ = 1677.37) (**6** − 2 H_2_O): C, 51.50; H, 5.04; N, 10.01%. Found: C, 51.18; H, 4.97; N, 9.84%. FT–IR (cm^−1^): 3073(w), 2914(w), 2854(w), 1682(w), 1614(m), 1587(m), 1542(s), 1477(w), 1428(s), 1363(s), 1280(m), 1193(w), 1133(w), 1101(w), 1052(w), 961(w), 807(w), 771(w), 730(m), 693(m), 567(w), 509(w), 475(w), 459(w), 448(m), 436(m), 423(w), 409(s).

#### 2.3.8. [Cu(**L^4^**)(AIPA)]*_n_*, c**7**

A mixture of Cu(OAc)_2_·H_2_O (0.020 g, 0.10 mmol), **L^4^** (0.032 g, 0.10 mmol), and H_2_AIPA (0.022 g, 0.10 mmol) in 5 mL of MeOH/H_2_O (1:1, *v*/*v*) solution was used. Yield: 0.049 g (79%). Anal. Calcd for C_28_H_21_CuN_5_O_7_ (*M*_W_ = 603.04): C, 55.81; H, 3.52; N, 11.63%. Found: C, 55.31 ; H, 3.89; N, 11.31%. FT–IR (cm^−1^): 3043(w), 2283(w), 1673(s), 1627(m), 1551(s), 1488(s), 1411(m), 1352(s), 1329(s), 1293(m), 1199(m), 1116(m), 1030(w), 937(w), 887(w), 861(w), 590(w), 538(w), 496(w), 454(w), 427(w), 416(m).

#### 2.3.9. {[Cu(**L^2^**)_0.5_(AIPA)]·MeOH}*_n_*, c**8**

A mixture of Cu(OAc)_2_·H_2_O (0.020 g, 0.10 mmol), **L^2^** (0.030 g, 0.10 mmol), and H_2_AIPA (0.022 g, 0.10 mmol) in 5 mL of MeOH/H_2_O (1:1, *v*/*v*) solution was used. Yield: 0.029 g (47%). Anal. Calcd for C_19_H_22_CuN_3_O_8_ (*M*_W_ = 483.94) (**8** + 1 H_2_O): C, 47.16; H, 4.58; N, 8.68%. Found: C, 47.02; H, 4.17; N, 8.91%. FT–IR (cm^−1^): 3841(w), 3736(w), 3677(w), 3053(w), 1714(m), 1653(m), 1595(m), 1541(s), 1485(m), 1418(s), 1374(s), 1324(m), 1288(m), 1197(m), 1174(m), 1134(m), 1621(m), 903(m), 779(m), 727(s), 706(m), 644(w), 540(w), 418(w), 481(m), 460(m), 442(m), 428(m), 414(m).

#### 2.3.10. {[Zn(**L^4^**)(AIPA)]·2H_2_O}*_n_*, c**9**

A mixture of Zn(OAc)_2_·4H_2_O (0.025 g, 0.10 mmol), **L^4^** (0.032 g, 0.10 mmol), and H_2_AIPA (0.022 g, 0.10 mmol) in 5 mL of MeOH/H_2_O (1:1, *v*/*v*) solution was used. Yield: 0.049 g (79%). Anal. Calcd for C_28_H_25_ZnN_5_O_9_ (*M*_W_ = 640.90): C, 52.47; H, 3.93; N, 10.93%. Found: C, 52.36; H, 3.78; N, 10.94%. FT–IR (cm^−1^): 3504(w), 3070(m), 2284(w), 1671(s), 1637(m), 1545(s), 1485(s), 1420(s), 1368(s), 1329(s), 1276(m), 1234(m), 1197(m), 1105(w), 1055(w), 1028(w), 940(w), 910(w), 886(w), 863(w), 810(m), 781(m), 719(m), 643(w), 602(w), 514(w), 452(w), 144(w).

#### 2.3.11. {[Zn(**L^2^**)(AIPA)]·2H_2_O}*_n_*, c**10**

A mixture of Zn(OAc)_2_·4H_2_O (0.025 g, 0.10 mmol), **L^2^** (0.030 g, 0.10 mmol), and H_2_AIPA (0.022 g, 0.10 mmol) in 5 mL of MeOH/H_2_O (1:1, *v*/*v*) solution was used. Yield: 0.029 g (47%). Anal. Calcd for C_26_H_29_ZnN_5_O_9_ (*M*_W_ = 620.91): C, 50.39; H, 4.72; N, 11.31%. Found: C, 50.18; H, 4.70; N, 11.06%. FT–IR (cm^−1^): 3484(m), 3243(m), 3119(m), 3039(m), 2945(m), 1662(s), 1630(m), 1576(s), 1553(s), 1488(s), 1420(s), 1365(s), 1330(m), 1287(s), 1221(m), 1140(m), 1100(w), 1064(w), 1031(w), 949(w), 909(w), 780(m), 700(m), 647(w), 542(m), 500(w), 457(w), 420(m).

### 2.4. Photodegradation Experiment

Tube 1 (blank), tube 2 (0.1 mL 30% H_2_O_2_), tube 3 (5 mg complex), and tube 4 (5 mg complex + 0.1 mL 30% H_2_O_2_) were prepared. To each tube was added 10 mL of a 50 ppm methyl blue (MB, C_37_H_27_N_3_Na_2_O_9_S_3_, Scheme S1 in Supplementary Materials) solution, which was prepared by diluting 50 mg MB with deionized water in a 1000 mL quantitative bottle. Each tube was then irradiated with the 365 nm UV light for 15, 30, 45, and 60 min, respectively; the absorption spectra were then measured. Tube 3 and tube 4 were first stirred in the dark for 15 min to determine the physical adsorption of the complex.

### 2.5. Single-Crystal X-ray Analysis

Single crystals of complexes **1** to **10** suitable for X-ray analysis were obtained from the hydrothermal reactions and their diffraction data were measured at 296 K using a Bruker AXS SMART APEX II CCD diffractometer, which was equipped with graphite monochromated MoK_α_ (λ_α_ = 0.71073 Å) radiation [10]. Data reductions were performed using the standard methods with well-established computational procedures and the empirical absorption corrections were carried out based on “multi-scan”. The atomic positions of some of the heavier atoms were located by the direct or Patterson method, followed by a series of alternating difference Fourier maps and least-square refinements to find the remaining atoms. Except those of the water molecules, the hydrogen atoms coordinated to the other atoms were added by using the HADD command in SHELXTL 6.1012 [11]. Basic information pertaining to the crystal parameters and structure refinement is summarized in Table 1.

## 3. Results and Discussion

### 3.1. Structural Descriptions

#### 3.1.1. Structure of {[Ni(**L^1^**)(3,5-PDA)(H_2_O)_3_]·2H_2_O}*_n_*, **1**

**Figure 3:**
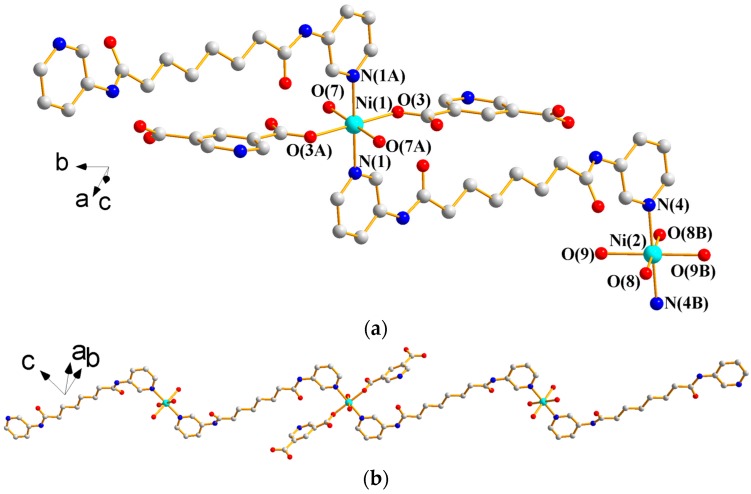
(**a**) Coordination environment about Ni(II) ions in **1**. Symmetry transformations used to generate equivalent atoms are (A) −x + 1, −y + 1, −z and (B) −x, −y − 1, −z + 3. (**b**) A drawing showing the 1D chain of **1**.

X-ray structural analysis reveals that complex **1** forms a 1D CP and crystallizes in the triclinic space group *P*ī with two independent halves of Ni(II) ions that occupy the inversion centers, one 3,5-PDA^2−^ ligand, three coordinated water molecules, and two lattice water molecules in the asymmetric unit. Figure 3a depicts a drawing showing the coordination environments about the Ni(II) ions, both of which adopt the distorted octahedral geometries. While the Ni(1) atom is coordinated by two pyridyl nitrogen atoms from two **L^1^** ligands [Ni(1)—N = 2.1214(15) Å] and four oxygen atoms from two 3,5-PDA^2−^ ligands and two water molecules [Ni(1)—O = 2.0338(11)–2.0788(11) Å], the Ni(2) atom is coordinated by two nitrogen atoms from two **L^1^** ligands [Ni(2)—N = 2.1189(15) Å] and four oxygen atoms from four water molecules [Ni(2)–O = 2.0342(11)–2.0890(12) Å]. Noticeably, the 3,5-PDA^2−^ ligands are coordinated to the metal ions in a monodentate fashion through one of the carboxylate oxygen atoms. The Ni(II) ions are bridged by the **L^1^** ligands to afford 1D chains (Figure 3b) that are supported by N–H---O (N---H = 2.01 and 2.05 Å; ∠N–H---O = 175.3 and 168.8°) hydrogen bonds originating from the amine hydrogen atoms to the oxygen atoms of the uncoordinated water molecules and 3,5-PDA^2−^ ligands, and O–H---O (O---H = 1.74–2.04 Å; ∠O–H---O = 153.2–175.5°) hydrogen bonds resulting from the interactions among coordinated water molecules, uncoordinated water molecules, 3,5-PDA^2−^ ligands, and **L^1^** ligands (see Table S1 and Figure S1). To our best knowledge, this type of *μ*_1_-κ^1^,κ^0^,κ^0^,κ^0^ bonding mode for the 3,5-PDA^2−^ ligands in CPs is unique.

#### 3.1.2. Structure of {[Ni_2_(**L^1^**)_2_(1,3,5-HBTC)_2_(H_2_O)_4_]·H_2_O}*_n_*, **2**

**Figure 4:**
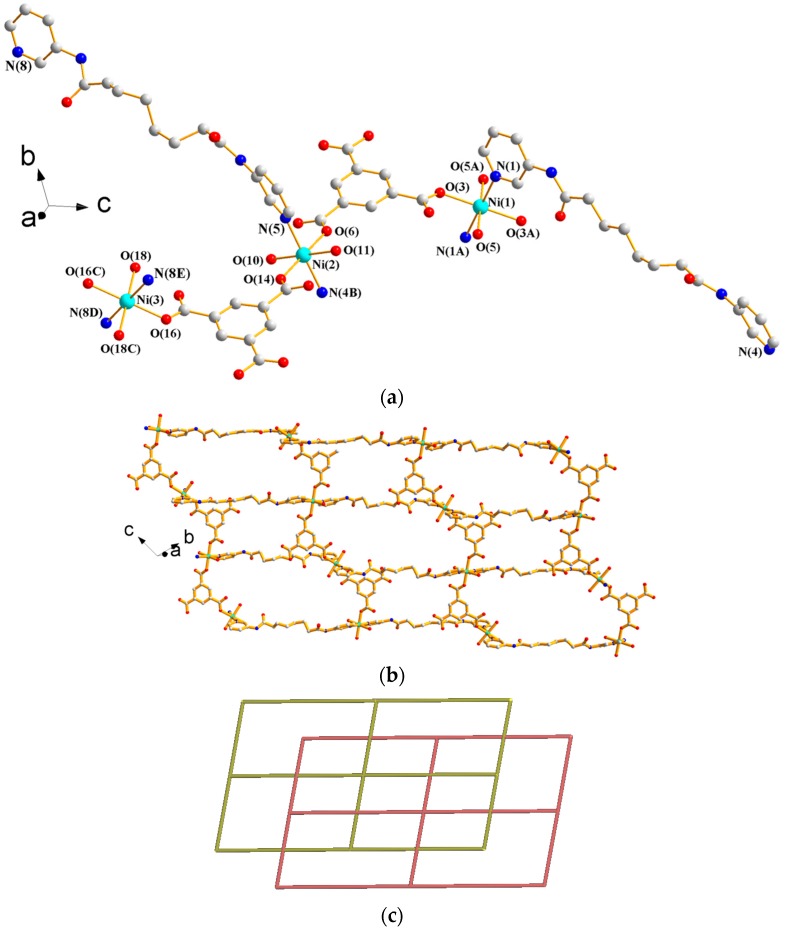
(**a**) Coordination environment about Ni(II) ions in **2**. Symmetry transformations used to generate equivalent atoms are (A) −x + 1, −y + 1, −z + 1, (B) −x, −y, −z + 1, (C) −x, −y, −z − 1, (D) −x + 1, −y + 1, −z − 1, and (E) x − 1, y − 1, z. (**b**) A drawing showing the 2D layer of **2**. (**c**) A drawing showing the two-fold interpenetration with the **sql** topology.

X-ray structural analysis reveals that complex **2** forms a 2D CP and crystallizes in the triclinic space group *P*ī. The asymmetric unit consists of one independent Ni(II) ion at the general position, two halves of an independent Ni(II) ion that occupy the inversion centers, two **L^1^** ligands, two 1,3,5-HBTC^2−^ ligands, four coordinated water molecules, and one lattice water molecule. Figure 4a depicts a drawing showing the coordination environments about the Ni(II) ions. All of the metal centers are six-coordinated by two pyridyl nitrogen atoms from two **L^1^** ligands [Ni–N = 2.091(2)–2.152(2) Å] and four oxygen atoms from two 1,3,5-HBTC^2−^ ligands [Ni–O = 2.0297(15)–2.1112(18) Å] and two coordinated water molecules [Ni–O = 2.0491(16)–2.0993(17) Å], resulting in distorted octahedral geometries. The Ni(II) ions are linked together by **L^1^** and 1,3,5-HBTC^2−^ ligands to form 2D layers, which stack along the *a* axis (see Figure S2 and Figure 4b). If the Ni(II) ions are defined as 4-connected nodes, the structure of **2** can be regarded as a two-fold interpenetrated and 4-connected net with the **sql** topology, determined using ToposPro [12] (see Figure 4c). The 2D layers are sustained by N–H---O (N---H = 2.22–2.35 Å; ∠N–H---O = 155.4–168.1°) hydrogen bonds to the oxygen atoms of the carboxylate and carbonyl oxygen atoms and O–H---O (O---H = 1.83–2.45 Å; ∠O–H---O = 125.3–174.8°) hydrogen bonds resulting from the interactions among coordinated water molecules, uncoordinated water molecules, 1,3,5-HBTC^2−^ ligands, and **L^1^** ligands (see Table S2 in Supplementary Materials).

#### 3.1.3. Structure of {[Ni(**L^2^**)(5-*tert*-IPA)(H_2_O)_2_]·2H_2_O}*_n_*, **3**

**Figure 5:**
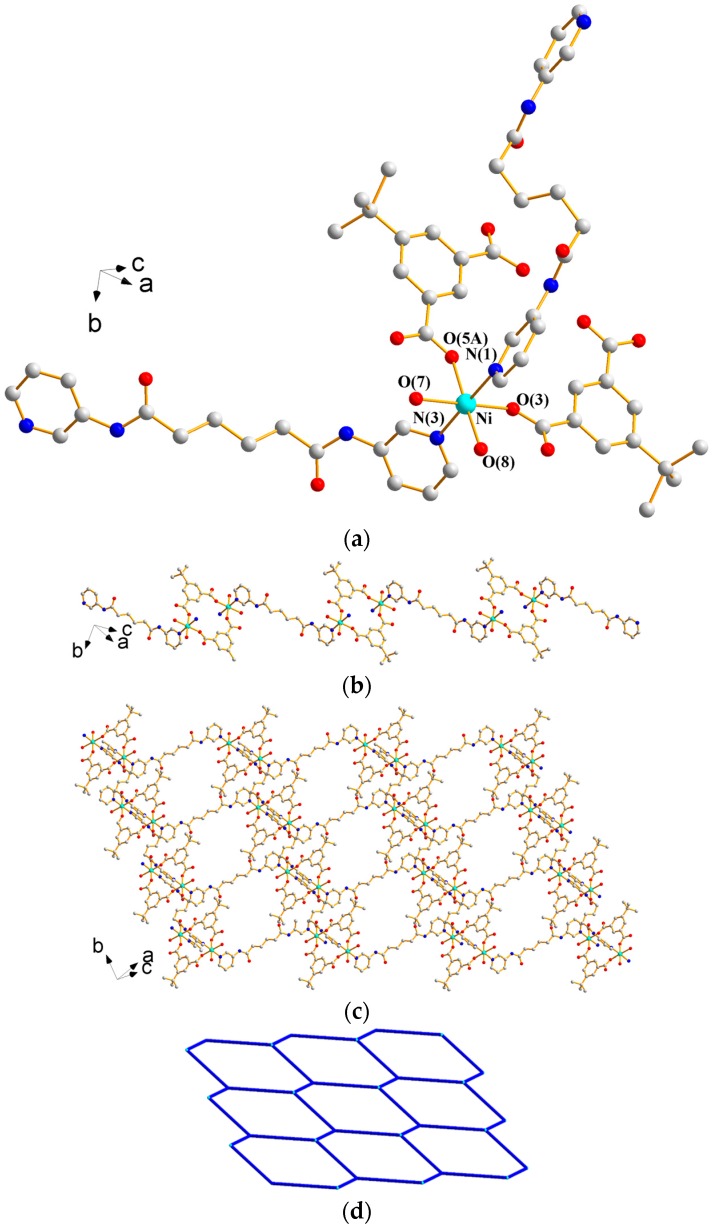
(**a**) Coordination environment about the Ni(II) ions in **3**. Symmetry transformations used to generate equivalent atoms are (A) −x + 1, −y + 1, −z. (**b**) A drawing showing the 1D chain of **3**. (**c**) A drawing showing the 2D layer of **3**. (**d**) A drawing showing the **hcp** topology of **3**.

X-ray structural analysis reveals that complex **3** forms a 2D CP and crystallizes in the triclinic space group *P*ī with one Ni(II) ion, one **L^2^** ligand, one 5-*tert*-IPA^2−^ ligand, two coordinated water molecules, and two lattice water molecules in the asymmetric unit. Figure 5a depicts a drawing showing the coordination environment about the Ni(II) ion, which is six-coordinated by two pyridyl nitrogen atoms from two **L^2^** ligands [Ni–N = 2.0910(18) and 2.0949(17) Å] and four oxygen atoms from two 5-*tert*-IPA^2−^ ligands [Ni–O = 2.0661(13) and 2.0878(12) Å] and two coordinated water molecules [Ni–O = 2.0641(16) and 2.1060(18) Å], resulting in a distorted octahedral geometry. Moreover, the Ni(II) ions are linked by the 5-*tert*-IPA^2−^ ligands and one independent **L^2^** ligand to afford 1D looped chains (see Figure 5b), which are further connected by the other independent **L^2^** ligands to form 2D layers (see Figure 5c), which stack along the *a* axis (see Figure S3 in Supplementary Materials). If the Ni(II) ions are defined as 3-connected nodes, the structure of **3** can be simplified as a 3-connected net with the **hcp** topology (Figure 5d). The 2D layers are supported by N–H---O (N---H = 1.97 and 2.07 Å; ∠N–H---O = 173.1 and 178.2°) hydrogen bonds to the uncoordinated water molecules and O–H---O (O---H = 1.86–2.35 Å; ∠O–H---O = 132.2–174.6°) hydrogen bonds resulting from the interactions among the coordinated water molecules, uncoordinated water molecules, 5-*tert*-IPA^2−^ ligands, and **L^2^** ligands (see Table S3 in Supplementary Materials).

#### 3.1.4. Structure of [Ni(**L^3^**)_1.5_(5-*tert*-IPA)]*_n_*, **4**

**Figure 6:**
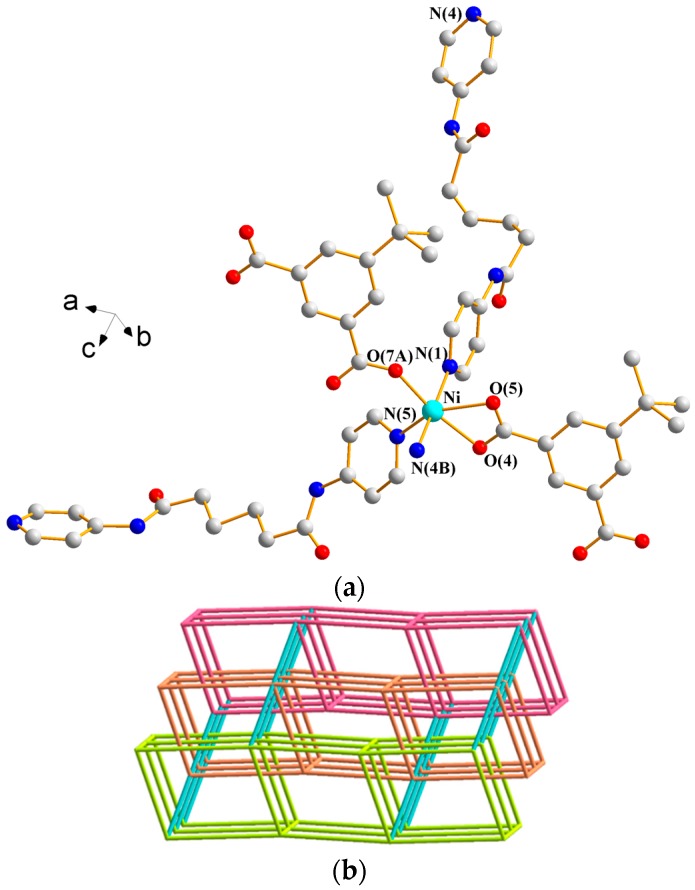
(**a**) Coordination environment about Ni(II) ions in **4**. Symmetry transformations used to generate equivalent atoms are (A) x + 1/2, y − 1/2, z; (B) x − 1/2, −y + 1/2, z + ½; (C) x − 1/2, y + 1/2, z; (D) x + 1/2, −y + 1/2, z − 1/2; and (E) −x + 1, y, −z + 1/2. (**b**) A drawing showing the 5-connected net with the (4^2^·6^7^·8)-**hxg**-d-5-*C*2/*c* topology.

X-ray structural analysis reveals that complex **4** forms a 3D CP and crystallizes in the monoclinic space group *C*2/*c*. The asymmetric unit consists of one Ni(II) ion, one half of an **L^3^** ligand, and one 5-*tert*-IPA^2−^ ligand. Figure 6a depicts a drawing showing the coordination environment about the Ni(II) ion, which is six-coordinated by three oxygen atoms from two 5-*tert*-IPA^2−^ ligands [Ni–O = 2.064(2)–2.263(2) Å] and three pyridyl nitrogen atoms from three **L^3^** ligands [Ni–N = 2.037(3)–2.092(3) Å], resulting in a distorted octahedral geometry. The Ni(II) ions are further linked by **L^3^** and 5-*tert*-IPA^2−^ ligands to form a 3D framework. Treating the Ni(II) cations as 5-connected nodes and the spacer ligands as linkers reveal a 3D net with the rare (4^2^·6^7^·8)-**hxg**-d-5-*C*2/*c* topology (see Figure 6b), which can be regarded as polycatenated nets cross-linked by the linkers. The (4^2^·6^7^·8)-**hxg**-d-5-*C*2/*c* net has been observed in two isostructural complexes {[Cu_2_(L4-4)X]_2_·CuX}*_n_* (X = Cl and Br; HL4-4 is 3,5-bis(4-pyridyl)-1H-pyrazole) that were obtained by reacting HL4-4 with corresponding copper salts (CuBr_2_/CuCl_2_), where the Cu(II) ions were reduced to monovalent Cu(I) in solvothermal conditions [13]. The 3D framework is supported by the N–H---O (N---H = 1.92 and 2.14 Å; ∠N–H---O = 174.4 and 157.8°) hydrogen bonds to the carboxylate oxygen atoms of the 5-*tert*-IPA^2−^ ligands (see Table S4 in Supplementary Materials).

#### 3.1.5. Structure of [Co(**L^1^**)(1,3,5-HBTC)(H_2_O)]*_n_*, **5**

**Figure 7:**
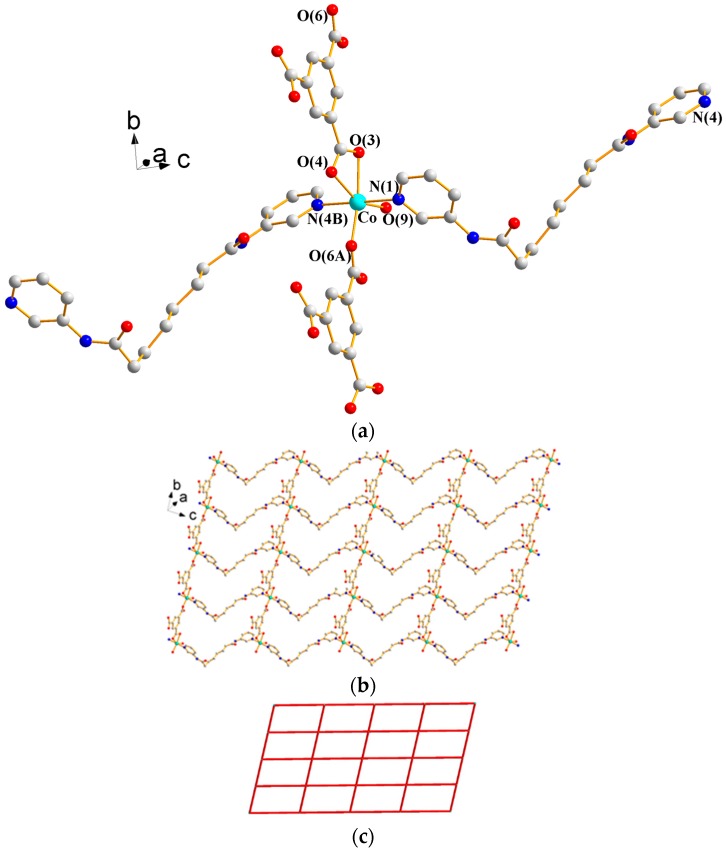
(**a**) Coordination environment about Co(II) ions in **5**. Symmetry transformations used to generate equivalent atoms are (A) x, y − 1, z; (B) x − 1, y, z − 1; (C) x, y + 1, z; and (D) x + 1, y, z + 1. (**b**) A drawing showing the 2D layer of **5**. (**c**) A drawing showing the **sql** topology of **5**.

X-ray structural analysis reveals that complex **5** forms a 2D CP and crystallizes in the triclinic space group *P*ī with one Co(II) ion, one **L^1^** ligand, one 1,3,5-HBTC^2−^ ligand, and one coordinated water molecule in the asymmetric unit. Figure 7a depicts a drawing showing the coordination environment about the Co(II) ion, which is six-coordinated by two pyridyl nitrogen atoms from two **L^1^** ligands [Co–N = 2.1561(15) and 2.1589(15) Å] and four oxygen atoms from two 1,3,5-HBTC^2−^ ligands [Co–O = 2.0141(13)–2.1804(13) Å] and one coordinated water molecule [Co–O = 2.0350(13) Å], resulting in a distorted octahedral geometry. Moreover, the Co(II) ions are linked together by the **L^1^** and 1,3,5-HBTC^2−^ ligands to afford 2D layers (Figure 7b), which stack along the *a* axis (see Figure S4). If the Co(II) ions are defined as 4-connected nodes, the structure of **5** can be regarded as a 4-connected net with the **sql** topology (Figure 7c). The 2D layers are sustained by N–H---O (N---H = 1.99 and 2.16 Å; ∠N–H---O = 179.3 and 174.7°) hydrogen bonds to the carbonyl and carboxylate oxygen atoms and O–H---O (O---H = 1.84–1.91 Å; ∠O–H---O = 155.9–174.4°) hydrogen bonds resulting from the interactions between coordinated water molecules and 1,3,5-HBTC^2−^ ligands (see Table S5 in Supplementary Materials).

#### 3.1.6. Structure of {[Co_3_(**L^1^**)_3_(1,3,5-BTC)_2_(H_2_O)_2_]·6H_2_O}*_n_*, **6**

**Figure 8:**
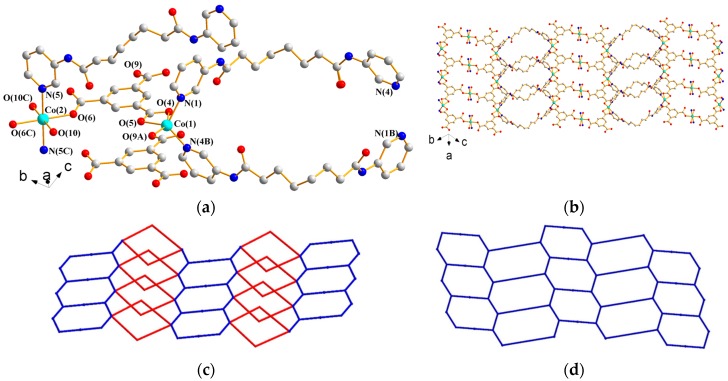
(**a**) Coordination environment about Co(II) ions in **6**. Symmetry transformations used to generate equivalent atoms are (A) x − 1, y, z; (B) −x + 1, −y, −z + 2; and (C) −x + 2, −y + 2, −z + 1. (**b**) A drawing showing the 2D layer of **6**. (**c**) A drawing showing the (4·8^5^)_2_(4)_2_(8^3^)_2_(8) topology of **6**. (**d**) A drawing showing the **hcp** topology of **6**.

X-ray structural analysis reveals that complex **6** forms a 2D CP and crystallizes in the triclinic space group *P*ī. The asymmetric unit consists of one independent Co(II) ion at the general position and a half of a Co(II) ion at the inversion center, one and a half L**^1^** ligands, one 1,3,5-BTC^3−^ ligand, one coordinated water molecule, and three co-crystallized water molecules. Figure 8a depicts a drawing showing the coordination environment about the Co(II) ions. The Co(1) atom adopts the distorted trigonal bipyramidal geometry (τ = 0.65) [14], which is coordinated by three oxygen atoms from two different 1,3,5-BTC^3−^ ligands [Co(1)–O = 1.957(3)–2.346(3) Å] and two pyridyl nitrogen atoms from two **L^1^** ligands [Co(1)–N = 2.055(3) and 2.061(3) Å], while the Co(2) atom adopts the distorted octahedral geometry, which is coordinated by two pyridyl nitrogen atoms from two **L^1^** ligands [Co(2)–N = 2.181(3) Å] and four oxygen atoms from two 1,3,5-BTC^3−^ ligands and two coordinated water molecules [Co(2)–O = 2.054(3)–2.112(3) Å]. The Co(II) ions are linked by **L^1^** ligands and 1,3,5-BTC^3−^ ligands to form 2D layers with loops (see Figure 8b), which stack along the *b* axis (see Figure S5 in Supplementary Materials). If the **L^1^** ligands are considered as 2-connected nodes, the structure of **6** can be simplified as a 2,2,3,4-connected (4·8^5^)_2_(4)_2_(8^3^)_2_(8) topology (as shown in Figure 8c), which can be further reduced to the **hcp** topology (as shown in Figure 8d) if the **L^1^** ligands are considered as linkers. Moreover, the 2D layers are supported by N–H---O (N---H = 2.00–2.26 Å; ∠N–H---O = 162.6–177.5°) hydrogen bonds to the carbonyl and carboxylate oxygen atoms and O–H---O (O---H = 1.69–2.28 Å; ∠O–H---O = 113.8–178.5°) hydrogen bonds resulting from the interactions among coordinated water molecules, uncoordinated water molecules, 1,3,5-BTC^3−^ ligands, and **L^1^** ligands (see Table S6).

#### 3.1.7. Structure of [Cu(**L^4^**)(AIPA)]*_n_*, **7**

**Figure 9:**
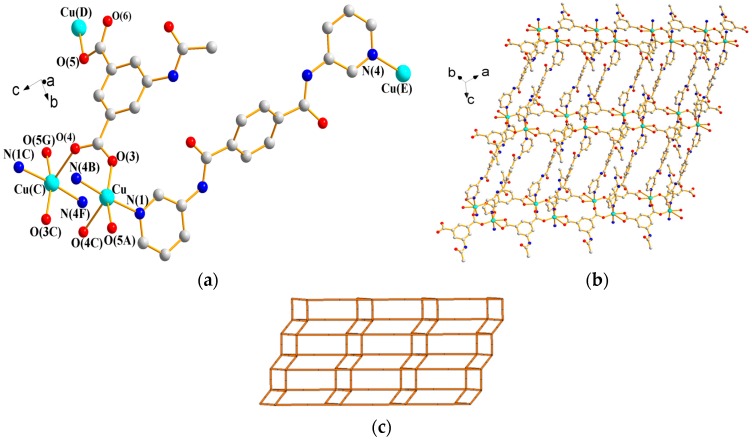
(**a**) Coordination environment about Cu(II) ions in **7**. Symmetry transformations used to generate equivalent atoms are (A) x − 1, y + 1, z; (B) x − 1, y, z + 1; (C) −x + 2, −y + 1, −z + 2; (D) x + 1, y − 1, z; (E) x + 1, y, z − 1; (F) –x + 3, −y + 1, −z + 1; and (G) –x + 3, −y, −z + 2. (**b**) A drawing showing the 2D layer of **7**. (**c**) A drawing showing the (4^2^·6^7^·8)(4^2^·6)-3,5L2 of **7**.

X-ray structural analysis reveals that complex **7** forms a 2D CP and crystallizes in the triclinic space group *P*ī with one Cu(II) ion, one **L^4^** ligand, and one AIPA^2−^ ligand in the asymmetric unit. Figure 9a depicts a drawing showing the coordination environment about the Cu(II) ions, which are five-coordinated by three oxygen atoms from three AIPA^2−^ ligands [Cu–O = 1.9220(17)–2.2115(18) Å] and two pyridyl nitrogen atoms from two **L^4^** ligands [Cu–N = 2.0410(2)–2.0610(2) Å], resulting in a distorted square pyramidal geometry (τ = 0.27). Moreover, the Cu(II) ions are linked by the AIPA^2−^ ligands to form dinuclear units [Cu---Cu = 4.202(2) Å], which are further linked by AIPA^2−^ and **L^4^** ligands to form 2D layers (Figure 9b), which stack along the *a* axis (see Figure S6). If the Cu(II) ions are considered as 5-connected nodes and the AIPA^2−^ ligands as 3-connected nodes, the structure of **7** can be simplified as a 3,5-connected net with the (4^2^·6^7^·8)(4^2^·6)-3,5L2 topology (Figure 9c). The 2D layers are supported by the N–H---O (N---H = 2.14–2.31 Å; ∠N–H---O = 150.7–166.7°) hydrogen bonds to the carbonyl and carboxylate oxygen atoms of AIPA^2−^ and **L^4^** ligands (see Table S7 in Supplementary Materials).

#### 3.1.8. Structure of {[Cu(**L^2^**)_0.5_(AIPA)]·MeOH}*_n_*, **8**

**Figure 10:**
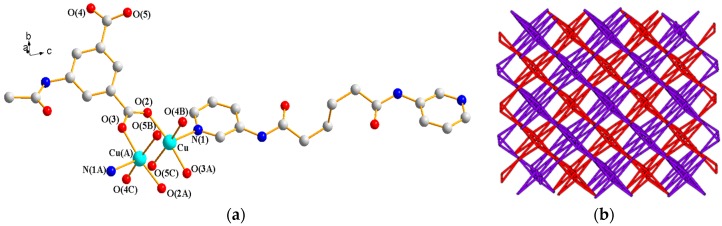

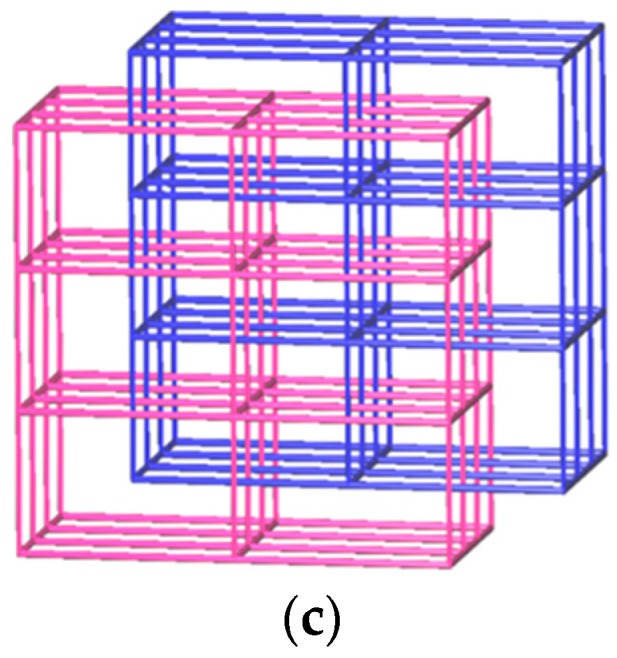
(**a**) Coordination environment about Cu(II) ions in **8**. Symmetry transformations used to generate equivalent atoms are (A) −x + 1, −y + 1, −z + 1; (B) x − 1/2, −y + 3/2, −z + 1; and (C) −x + 3/2, y − 1/2, z. (**b**) A drawing showing the 2-fold interpenetrated net with the (3^2^·6^2^·7^2^)(3^4^·4^6^·6^4^·7)-sqc493 topology. (**c**) A drawing showing the 2-fold interpenetrated net **pcu** topology of **8**.

X-ray structural analysis reveals that complex **8** forms a 3D CP and crystallizes in the orthorhombic space group *P*bca. The asymmetric unit consists of one Cu(II) ion, a half of **L^2^** ligand, one AIPA^2−^ ligand, and one lattice methanol molecule. Figure 10a depicts a drawing showing the coordination environments about the Cu(II) ions, which are five-coordinated by four oxygen atoms from four AIPA^2−^ ligands [Cu–O = 1.9600(2)–1.9780(2) Å] and one pyridyl nitrogen atom from one **L^2^** ligand [Cu–N = 2.1660(2) Å], resulting in a distorted square pyramidal geometry (τ = 0.01). The metal atoms are linked by the AIPA^2−^ ligands to form dinuclear units [Cu---Cu = 2.6431(6) Å], which are further connected by AIPA^2−^ and **L^2^** ligands to form a 3D framework. If the Cu(II) ions are considered as 6-connected nodes and the AIPA^2−^ ligands as 4-connected nodes and the **L^2^** ligands as linkers, the structure of **8** can be simplified as a 4,6-connected, 2-fold interpenetrated net with the (3^2^·6^2^·7^2^)(3^4^·4^6^·6^4^·7)-sqc493 topology (Figure 10b). Furthermore, treating the dinuclear Cu(II) units as 6-connected nodes affords a 2-fold interpenetrated net with **pcu** topology (Figure 10c). N–H---O (N–--H = 2.01 and 2.18 Å; ∠N–H---O = 171.8–173.7°) hydrogen bonds to the carbonyl oxygen atoms of AIPA^2−^ ligands and the methanol oxygen atoms and O–H---O (O---H = 2.00 Å; ∠O–H---O = 161.2°) hydrogen bonds originating from methanol to the carbonyl oxygen atoms of AIPA^2−^ ligands are found in the 3D framework (see Table S8 in Supplementary Materials).

#### 3.1.9. Structure of {[Zn(**L^4^**)(AIPA)]·2H_2_O}*_n_*, **9**

**Figure 11:**
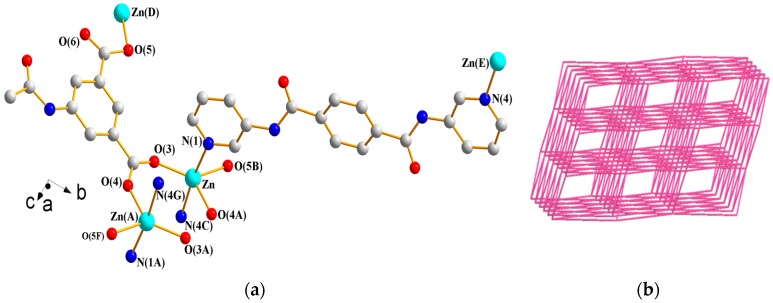

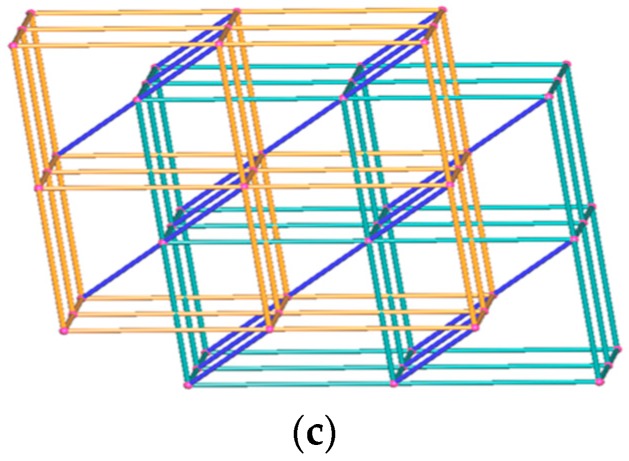
(**a**) Coordination environment about Zn(II) ions in **9**. Symmetry transformations used to generate equivalent atoms are (A) −x + 3/2, −y + 1/2, −z + 1; (B) −x + 3/2, y + 1/2, −z + 1/2; (C) x + 1/2, −y + 3/2, z + 1/2; (D) −x + 3/2, y − 1/2, −z + 1/2; (E) x − 1/2, −y + 3/2, z − 1/2; (F) x, –y, z + 1/2; and (G) −x + 1, y − 1, −z + 1/2. (**b**) A drawing showing the (4^24^·6^4^)-8T2 topology of **9**. (**c**) A drawing showing two-fold interpenetration of **pcu**-type nets cross-linked by the linkers.

X-ray structural analysis reveals that complex **9** forms a 3D CP and crystallizes in the monoclinic space group *C*2/*c* with one Zn(II) ion, one **L^4^** ligand, one AIPA^2−^ ligand, and two lattice water molecules in the asymmetric unit. Figure 11a depicts a drawing showing the coordination environments about the Zn(II) ions. Each of the symmetry-related Zn(II) ions is five-coordinated by three oxygen atoms from three AIPA^2−^ ligands [Zn–O = 1.9840(2)–2.0720(2) Å] and two pyridyl nitrogen atoms from two **L^4^** ligands [Zn–N = 2.1700(2)–2.1890(2) Å], resulting in a distorted trigonal bipyramidal geometry (τ = 0.61). The metal ions are linked by two AIPA^2−^ ligands to form dinuclear units [Zn---Zn = 3.979(2) Å], which are further linked by **L^4^** and AIPA^2−^ ligands to form a 3D framework. Treating the dinuclear Zn(II) units as 8-connected nodes and the spacer ligands as linkers reveal the presence of a self-catenated 3D net with the (4^24^·6^4^)-8T2 topology (Figure 11b). A closer investigation reveals that the topological structure can be viewed as a two-fold interpenetration of **pcu**-type nets cross-linked by the linkers, as shown in Figure 11c. Moreover, the 3D framework is supported by N–H---O (N---H = 2.13–2.52 Å; ∠N–H---O = 127.7–156.6°) hydrogen bonds to the carbonyl oxygen atoms of AIPA^2−^ ligands and the water oxygen atoms and O–H---O (O---H = 1.83–2.18 Å; ∠O–H---O = 125.3–165.0°) hydrogen bonds originating from water molecules to the carbonyl oxygen atoms of AIPA^2−^ ligands, carbonyl oxygen atoms of **L^4^** ligands, and the water oxygen atoms (see Table S9 in Supplementary Materials).

#### 3.1.10. Structure of {[Zn(**L^2^**)(AIPA)]·2H_2_O}*_n_* **10**

**Figure 12:**
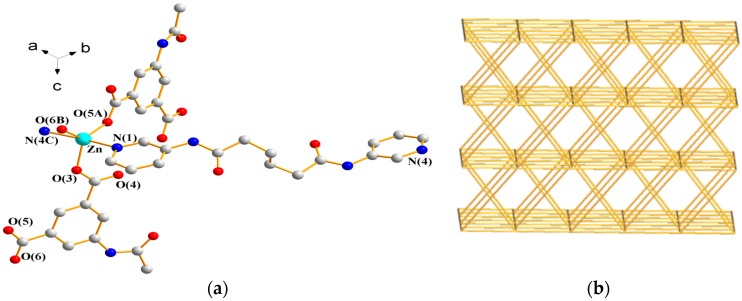
(**a**) Coordination environment about Zn(II) ions in **10**. Symmetry transformations used to generate equivalent atoms are (A) −x + 1, y + 1/2, −z + 3/2; (B) x, −y + 1/2, z − 1/2; (C) x + 1, y − 1, z; (D) −x + 1, y − 1/2, −z + 3/2; (E) x, −y + 1/2, z + 1/2; (F) x − 1, y + 1, z; (G) –x, y − 3/2, −z + 3/2; (H) −x + 1, −y, −z + 2; and (I) x + 1, −y + 3/2, z + 1/2. (**b**) A drawing showing the (4^4^·6^10^·8)-**mab** topology of **10**.

X-ray structural analysis reveals that complex **10** forms a 3D CP and crystallizes in the monoclinic space group *P*2_1_/*c*. The asymmetric unit consists one Zn(II) ion, one **L^2^** ligand, one AIPA^2−^ ligand, and two lattice water molecules. Figure 12a depicts a drawing showing the coordination environment about the Zn(II) ion, which is five-coordinated by three oxygen atoms from three AIPA^2−^ ligands [Zn–O = 1.9693(6)–2.0206(6) Å] and two pyridyl nitrogen atoms from two **L^2^** ligands [Zn–N = 2.1918(21) and 2.1615(23) Å], resulting in a distorted trigonal bipyramidal geometry (τ = 0.67). The metal atoms are linked by two AIPA^2−^ ligands to form dinuclear units [Zn---Zn = 3.8970(5) Å], which are further linked by **L^2^** and AIPA^2−^ ligands to form a 3D framework. If the dinuclear Zn(II) units are defined as 6-connected nodes and the spacer ligands as linkers, the structure of **10** can be regarded as an underlying self-catenated net with the (4^4^·6^10^·8)-**mab** topology (Figure 12b). It is noted that the **mab** net is closely related to the self-catenated net **roa**. Both of the nets have the same Schläfli symbol (4^4^·6^10^·8) but differ in the vertex symbols, which are 4·4·4·4·6_4_·6_4_·6_5_·6_5_·6_5_·6_5_·6_11_·6_11_·6_11_·6_11_* and 4·4·4·4·6_2_·6_2_·6_5_·6_5_·6_5_·6_5_·6_5_·6_5_·6_5_·6_5_*, respectively. The 3D framework is sustained by N–H---O (N---H = 2.01–2.12 Å; ∠N–H---O = 149.3–178.0°) hydrogen bonds to the carbonyl and carboxylate oxygen atoms of AIPA^2−^ ligands and the water oxygen atoms and O–H---O (O---H = 2.18–2.48 Å; ∠O–H---O = 116.8–170.6°) hydrogen bonds originating from water molecules to the carboxylate oxygen atoms of AIPA^2−^ ligands and carbonyl oxygen atoms of **L^2^** ligands (see Table S10).

The solvent-accessible volumes of complexes **1** to **10** have been calculated using PLATON [15], which are 1.1, 0.6, 6.7, 0, 0, 14.9, 0, 6.6, 7.9, and 10.1% of each of their corresponding unit cell volumes, respectively, indicating small or no porosity.

### 3.2. Ligand Conformations and Bonding Modes

The descriptor that has been proposed to establish the ligand conformations of the bpba ligands is applicable for **L^1^**–**L^4^** [5]: (a) If the C–C–C–C torsion angle (θ) of the backbone carbon atoms is 180 ≥ θ > 90° and 0 ≤ θ ≤ 90°, **L^1^**–**L^3^** adopt the A and G conformations, respectively. (b) On the basis of the relative orientation of the C=O (or N–H) groups, each of the **L^1^**–**L^4^** ligands adopt the cis or trans arrangement. (c) Due to the different orientations of the pyridyl nitrogen atom positions, three more orientations—*anti-anti*, *syn-anti*, and *syn-syn*—can also be imposed on **L^1^**, **L^2^**, and **L^4^**. Accordingly, the ligand conformations of complexes **1** to **10** are assigned and listed in Table 2, which also shows the various bonding modes for the polycarboxylate ligands. The diverse ligand conformations and bonding modes shown in Table 2 indicate that **L^1^**–**L^4^** and the polycarboxylate ligands are flexible and can be adjusted to fit the stereochemical requirements for forming the structures of complexes **1** to **10**, which presumably result from the maximization of their intra and intermolecular forces.

### 3.3. Structural Comparisons

The structural difference between **1** and **2** is presumably due to the different identities of the dicarboxylate ligands. Structural comparisons of complexes **3** and **4** reveal that ligand isomerism (the donor atom position of the bpba ligand) is important in determining the structural diversity, resulting in a 2D layer with the **hcp** topology and a 3D framework with the (4^2^·6^7^·8)-**hxg**-d-5-C2/c topology, respectively. The different metal/ligand ratios used for complexes **5** and **6** are also important in determining the structural type, giving a 2D layer with the **sql** topology and a 2D layer with the **hcp** topology, respectively. Moreover, a comparison of the structures of complexes **7**–**10** shows that the metal centers and flexibility of the ligands significantly affect the structural diversity. The metal centers are Cu(II) in 7 and Zn(II) in **9**, affording a 2D layer with the **sql** topology and a self-catenated 3D framework with the (4^24^·6^4^)-8T2 topology, respectively. Similarly, complexes **8** and **10** display a 2-fold interpenetrated 3D framework with the **pcu** topology and a self-catenated 3D framework with the (4^4^·6^10^·8)-**mab** topology, respectively. It is also shown that with the same bpba and AIPA^2−^ ligands, the CPs with Zn(II) ions prefer to form 3D self-catenated frameworks (7 vs. **9** and **8** vs. **10**), which can be ascribed to the formation of the distorted trigonal bipyramidal geometries in complexes **9** and **10**, rather than the distorted square pyramidal geometries in complexes **7** and **8**. Structural differences in the pairs of **7** and **8**, and **9** and **10** are presumably due to the different flexibility between **L^4^** and **L^2^** ligands. Structural comparisons of **1**–**10** indicate that the identity of the dicarboxylate ligands, the ligand isomerism and flexibility of the bpba ligands, the metal/ligand ratio, and the nature of the metal centers play important roles in determining the structural diversity of the divalent coordination polymers.

### 3.4. Luminescent Properties

Luminescent d^10^ metal complexes have the ability to enhance, shift, and quench emissions of organic ligands, which are of great interest due to the potential applications of these complexes in areas such as sensors and displays. The luminescent properties of **9** and **10** were thus investigated. Table 3 summarizes the UV–VIS and luminescent properties of complexes **9** and **10** and the free organic ligands **L^2^**, **L^4^**, and H_2_AIPA. Figures S7–S11 depict the corresponding excitation/emission spectra, which were measured in solid state at room temperature. The spectra of **L^2^**, **L^4^**, and H_2_AIPA show emissions in the range of 415–425 nm, which may be ascribed to the intraligand (IL) n → π* or π → π* transitions. The red shifts of **9** with respect to the free organic ligands can be ascribed to the different ligand conformations and coordination modes adopted by the organic ligands and the formation of different structural types. It is well known that the Zn(II) ion is hardly susceptible to oxidation or reduction and it is thus not possible that the emissions of **9** and **10** are due to ligand-to-metal charge transfer (LMCT) or metal-to-ligand charge transfer (MLCT). Therefore, these emissions may be attributed to intraligand or ligand-to-ligand charge transfer (LLCT) [16].

### 3.5. Photocatalytic Properties

The photocatalytic role of CPs in the detoxification of water from organic pollutants is a subject of current interest [17,18]. However, only a few bpba-based CPs have exhibited plausible photocatalytic properties [6,19]. It was proposed that in photocatalysis, the hydroxyl radical (OH·) is the major oxidant in charge of the heterogeneous oxidation process, which decomposes the organic dye with good efficiency. Due to the easy preparation and applicable stability in water, the photocatalytic effects of complexes **9** and **10** with the degradation of a dye were investigated. Methyl blue (MB) was selected as a model of dye contaminant and the experiments were carried out in 30 wt % H_2_O_2_ under 365 nm UV light.

**Figure 13:**
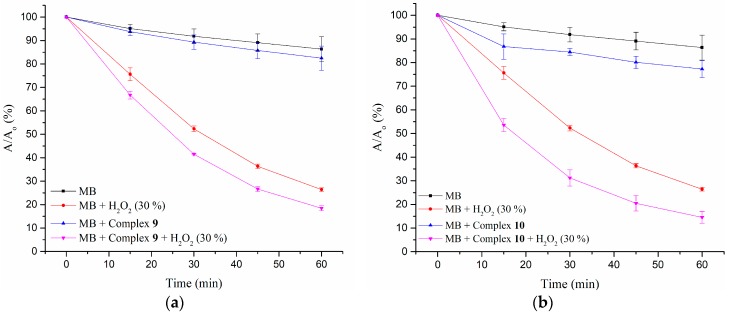
The changes in A/A_0_ of methyl blue (MB) solutions versus irradiation time for complexes (**a**) **9** and (**b**) **10**.

The changes in A/A_0_ of MB solutions versus irradiation time for complexes **9** and **10** are shown in Figure 13a,b, respectively, wherein A_0_ is the initial absorption of the MB solution and A is the absorption at the reaction time t (min). Time-dependent absorption spectra of the MB solution under 365 nm UV light are provided as supplementary material in Figures S12–S29. The percentage of MB (pmb) remaining in solution was evaluated according to pmb = (A_0_ − A_t_)/A_0_ × 100%, while the degradation efficiency (de) was calculated as de = pmb (t = 0)–pmb (t = 60) (see Table S11 in Supplementary Materials). Accordingly, the enhanced de due to the complexes with the participation of H_2_O_2_ is 8(1) and 12(2)% for **9** and **10**, respectively. It is clearly shown that both complexes exhibit a photocatalytic effect toward MB degradation. Moreover, the PXRD patterns of complexes **9** and **10** after the photocatalytic reactions have been measured. As shown in Figure S30, no obvious changes are observed, indicating their structural integrity and suitability for being used as catalysts in the photocatalytic reaction system. For comparison, it is noted that the 3-fold interpenetrating coordination polymer Zn(bdc)(L’)·solvents (L’ = N^4^,N^4^′-di(pyridin-4-yl)biphenyl-4,4′-dicarboxamide, H_2_bdc = terephthalic acid), which adopts a self-catenated net with the **hxg**-d-4-Fddd topology, shows photocatalytic activity under visible light with the degradation of rhodamine B (RhB) [17].

## 4. Conclusions

The synthesis and structural characterization of ten divalent CPs based on bpba and polycarboxylate ligands have been successfully accomplished, which show 1D, 2D, and 3D structures. Complex **1** is a 1D chain and **2** and **5** are 2D layers with the **sql** topology, while **3** and **6** are 2D layers with the **hcp** topology and **4** displays a 3D self-catenated framework with the rare (4^2^·6^7^·8)-**hxg**-d-5-*C*2/*c* topology. Complex **7** forms a 2D layer with the (4^2^·6^7^·8)(4^2^·6)-3,5L2 topology and **8** is a 2-fold interpenetrated 3D framework with the **pcu** topology, while **9** and **10** display 3D self-catenated frameworks with the (4^24^·6^4^)-8T2 and the (4^4^·6^10^·8)-**mab** topologies, respectively. In addition to the nature of the dicarboxylate ligands, we have shown that the identity of the metal ions, the isomerism and flexibility of the bpba ligands, and the metal to ligand ratio play important roles in determining the structural diversity of these divalent CPs. With the same bpba and AIPA^2−^ ligands, the CPs with Zn(II) ions prefer to form the 3D self-catenated frameworks. We have also shown that both complexes **9** and **10** display luminescent properties and can be used as catalysts to accelerate the photodegradation of organic dyes.

## Figures and Tables

**Table 1:** Crystal data for complexes **1** to **10**.

**Complex**	**1**	**2**	**3**	**4**	**5**
Formula	C_25_H_35_NiN_5_O_11_	C_54_H_62_Ni_2_N_8_O_21_	C_28_H_38_NiN_4_O_10_	C_36_H_39_NiN_6_O_7_	C_27_H_28_CoN_4_O_9_
Formula weight	640.29	1276.53	649.33	726.44	611.46
Crystal system	Triclinic	Triclinic	Triclinic	Monoclinic	Triclinic
Space group	*P*ī	*P*ī	*P*ī	*C*2/*c*	*P*ī
a, Å	10.2217(2)	9.2151(1)	11.7614(2)	12.9109(14)	9.6229(1)
b, Å	12.1140(2)	15.2396(2)	12.0360(2)	16.2298(17)	10.2784(1)
c, Å	13.1648(3)	20.1781(2)	12.2961(2)	33.736(4)	14.7068(2)
α, °	67.612(1)	105.765(1)	78.548(1)	90	87.707(1)
β, °	76.879(1)	96.500(1)	67.752(1)	95.395(7)	89.509(1)
γ, °	70.475(1)	90.244(1)	86.067(1)	90	73.917(1)
V, Å^3^	1411.27(5)	2707.76(6)	1578.94(5)	7037.8(13)	1396.56(3)
Z	2	2	2	8	2
d_calc_, mg/m^3^	1.507	1.566	1.366	1.371	1.454
F(000)	672	1332	684	3048	634
µ(Mo K_α_), mm^−1^	0.756	0.786	0.674	0.609	0.674
Range (2θ) for data collection	3.36 to 56.68	3.92 to 56.72	3.46 to 56.59	4.14 to 56.92	4.12 to 56.56
Independent reflections	7003	13388	7815	8791	6910
	[R(int) = 0.0355]	[R(int) = 0.0253]	[R(int) = 0.0245]	[R(int) = 0.0337]	[R(int) = 0.0185]
Data/restraints/parameters	7003/0/412	13388/1369/819	7815/0/395	8791/808/484	6910/0/382
Quality-of-fit indicator ^c^	1.076	1.062	1.037	1.067	1.053
Final R indices [I > 2σ(I)] ^a,b^	R1 = 0.0329	R1 = 0.0472	R1 = 0.0401	R1 = 0.0666	R1 = 0.0353
	wR2 = 0.0731	wR2 = 0.1282	wR2 = 0.1018	wR2 = 0.1864	wR2 = 0.0925
R indices (all data)	R1 = 0.0473	R1 = 0.0756	R1 = 0.0533	R1 = 0.0955	R1 = 0.0426
	wR2 = 0.0793	wR2 = 0.1460	wR2 = 0.1096	wR2 = 0.2044	wR2 = 0.0962
**Complex**	**6**	**7**	**8**	**9**	**10**
Formula	C_72_H_88_Co_3_N_12_O_26_	C_28_H_21_CuN_5_O_7_	C_19_H_20_CuN_3_O_7_	C_28_H_25_ZnN_5_O_9_	C_26_H_29_ZnN_5_O_9_
Formula weight	1714.33	603.04	465.92	640.90	620.91
Crystal system	Triclinic	Triclinic	Orthorhombic	Monoclinic	Monoclinic
Space group	*P*ī	*P*ī	*P*bca	*C*2/*c*	*P*2_1_/*c*
a, Å	10.0136(2)	9.7425(2)	12.8022(2)	26.4979(10)	12.5193(1)
b, Å	11.9834(3)	9.9983(2)	14.5368(2)	12.7299(5)	12.8079(2)
c, Å	17.2930(4)	15.1361(2)	20.5531(2)	17.1560(6)	16.8353(2)
α, °	98.375(2)	81.021(1)	90	90	90
β, °	90.027(1)	84.597(1)	90	108.868(2)	91.488(1)
γ, °	95.361(1)	61.683(1)	90	90	90
V, Å^3^	2043.79(8)	1281.71(4)	3824.99(9)	5476.0(4)	2698.56(6)
Z	1	2	8	8	4
d_calc_, mg/m^3^	1.393	1.563	1.618	1.555	1.528
F(000)	893	618	1920	2640	1288
µ(Mo K_α_), mm^−1^	0.685	0.911	1.192	0.962	0.973
Range(2θ) for data collection	3.89 to 52.00	4.66 to 56.64	4.68 to 56.64	3.25 to 56.66	3.26 to 56.58
Independent reflections	8041	6365	4760	6783	6539
	[R(int) = 0.0411]	[R(int) = 0.0551]	[R(int) = 0.0486]	[R(int) = 0.0609]	[R(int) = 0.0193]
Data/restraints/parameters	8041/0/568	6265/0/374	4760/0/283	6783/0/397	6539/0/370
Quality-of-fit indicator ^c^	1.053	1.056	1.021	1.024	1.023
Final R indices [I > 2σ(I)] ^a,b^	R1 = 0.0660,	R1 = 0.0453,	R1 = 0.0438,	R1 = 0.0521,	R1 = 0.0399,
	wR2 = 0.1807	wR2 = 0.1040	wR2 = 0.0997	wR2 = 0.0930	wR2 = 0.1144
R indices (all data)	R1 = 0.0907,	R1 = 0.0714,	R1 = 0.0724,	R1 = 0.1033,	R1 = 0.0510,
	wR2 = 0.1980	wR2 = 0.1150	wR2 = 0.1129	wR2 = 0.1090	wR2 = 0.1219

^a^ R_1_ = Σ||F_o_| − |F_c_||/Σ|F_o_|; ^b^ wR_2_ = [Σw(F_o_^2^ − F_c_^2^)^2^/Σw(F_o_^2^)^2^]^1/2^. w = 1/[σ^2^(F_o_^2^) + (ap)^2^ + (bp)], p = [max(F_o_^2^ or 0) + 2(F_c_^2^)]/3. a = 0.0318, b = 0.4608, **1**; a = 0.0655, b = 2.0327, **2**; a = 0.0533, b = 0.9980, **3**; a = 0.1035, b = 14.0450, **4**; a = 0.0473, b = 0.7901, **5**; a = 0.1153, b = 2.1116, **6**; a = 0.0495, b = 0.6944, **7**; a = 0.0421, b = 6.0326, **8**; a = 0.0401, b = 3.4024, **9**; a = 0.0705, b = 1.4721, **10**; ^c^ quality-of-fit = [Σw(|F_o_^2^| − |F_c_^2^|)^2^/N_observed_ − N_parameters_ )]^1/2^.

**Table 2:** Ligand conformation of **L^1^** to **L^4^** and bonding modes of the polycarboxylate ligands in **1**–**10**.

Complex	Ligand conformation	Bonding mode
{[Ni(**L^1^**)(3,5-PDA)(H_2_O)_3_]·2H_2_O}*_n_*, **1**	AAAAA trans *syn-syn*	*μ*_1_-κ^1^,κ^0^,κ^0^,κ^0^
{[Ni_2_(**L^1^**)_2_(1,3,5-HBTC)_2_(H_2_O)_4_]·H_2_O}*_n_*, **2**	AAAAA trans *anti-syn* AGAGA trans *syn-anti*	*μ*_2_-κ^1^,κ^0^,κ^1^,κ^0^,κ^0^,κ^0^
{[Ni(**L^2^**)(5-*tert*-IPA)(H_2_O)_2_]·2H_2_O}*_n_*, **3**	AAA trans *anti-anti* GAG trans *syn-anti*	*μ*_2_-κ^1^,κ^0^,κ^1^,κ^0^
[Ni(**L^3^**)_1.5_(5-*tert*-IPA)]*_n_*, **4**	AAA trans GAG trans	*μ*_2_-κ^1^,κ^0^,κ^1^,κ^0^
[Co(**L^1^**)(1,3,5-HBTC)(H_2_O)]*_n_*, **5**	GAAAA cis *anti-syn*	*μ*_2_-κ^1^,κ^0^,κ^1^,κ^1^,κ^0^,κ^0^
{[Co_3_(**L^1^**)_3_(1,3,5-BTC)_2_(H_2_O)_2_]·6H_2_O}*_n_*, **6**	AAGGA cis *syn-syn*	*μ*_3_-κ^1^,κ^0^,κ^1^,κ^0^,κ^1^,κ^1^
	AGAGA trans *syn-syn*	
[Cu(**L^4^**)(AIPA)]*_n_*, **7**	trans *syn-syn*	*μ*_3_-κ^0^,κ^1^,κ^1^,κ^1^
{[Cu(**L^2^**)_0.5_(AIPA)]·MeOH}*_n_*, **8**	GAG trans *anti-anti*	*μ*_4_-κ^1^,κ^1^,κ^1^,κ^1^
{[Zn(**L^4^**)(AIPA)]·2H_2_O}*_n_*, **9**	trans *anti-anti*	*μ*_3_-κ^0^,κ^1^,κ^1^,κ^1^
{[Zn(**L^2^**)(AIPA)]·2H_2_O}*_n_*, **10**	GAG trans *anti-anti*	*μ*_3_-κ^0^,κ^1^,κ^1^,κ^1^

**Table 3:** The absorption, excitation, and emission wavelengths (nm) of **L^2^**, **L^4^**, H_2_AIPA, and complexes **9** and **10** in the solid state.

Complex	λ_abs_ (nm)	λ_ex_ (nm)	λ_em_ (nm)
**L^2^**	300	370	415
**L^4^**	341	330/365	420
H_2_AIPA	323	336	425
9	340	340/400	460
10	322	325/350	415

## Supplementary Materials

Supplementary materials can be found at www.mdpi.com/2073-4360/9/12/691/s1. Crystallographic data for **1**–**10** have been deposited with the Cambridge Crystallographic Data Centre, CCDC No. 1570989-1570998.
