# Supplementary material for: Metal and Ligand Effects on the Construction of Divalent Coordination Polymers Based on bis-Pyridyl-bis-amide and Polycarboxylate Ligands
Source: Polymers (Basel). 2017 Dec 8;9(12):691. doi: 10.3390/polym9120691 (PMC6418995; doi:10.3390/polym9120691)

Metal and Ligand Effect on the Construction of Divalent Coordination Polymers Based on Bis-pyridyl-bis-amide and Polycarboxylate Ligands

Miao-Ning Chang, Xiang-Kai Yang,

Pradhumna Mahat Chhetri and Jhy-Der Chen*

Department of Chemistry, Chung-Yuan Christian University, Chung-Li, Taiwan, R.O.C.

Table S1. H-bonding parameters in compound **1**.

D-H	D-H(Å)	H…A (Å)	<DH…A(º)	D…A (Å)	A	Symmetry
N2-H2A	0.86	2.051	168.76	2.899	O5	[-x+1, -y+1, -z]
N3-H3A	0.86	2.004	175.27	2.862	O10	[-x, -y+1, -z]
O7-H7C	0.768	1.946	167.42	2.701	O2	[x, y+1, z-1]
O7-H7D	0.912	1.739	162.64	2.623	O4	[-x+1, -y+2, -z-1]
O8-H8C	0.848	1.948	161.54	2.766	O1	[x, y-1, z+1]
O8-H8D	0.855	1.748	175.45	2.601	O11	[x, y-1, z+1]
O9-H9C	0.825	2.037	153.17	2.798	O5	[x, y, z+1]
O9-H9D	0.847	1.984	174.4	2.827	N5	[-x, -y+1, -z+1]
O10-H10C	0.827	1.987	172.2	2.809	O6	
O10-H10D	0.801	2.036	167.19	2.822	O4	[-x+1, -y+1, -z-1]
O11-H11C	0.858	1.822	174.47	2.677	O6	[x, y+1, z]
O11-H11D	0.807	2.015	168.28	2.811	O7	

Table S2. H-bonding parameters in compound **2**.

D-H	D-H (Å)	H…A (Å)	<DH…A(º)	D…A (Å)	A	Symmetry
O5-H5C	0.85	2.154	136.5	2.832	O13	[-x+2, -y+1, -z+1]
O5-H5D	0.85	1.867	149.29	2.633	O4	
O9-H9D	0.85	1.834	164.74	2.663	O17	[-x+2, -y+1, -z+1]
O10-H10D	0.85	1.94	146.22	2.689	O7	
O10-H10E	0.85	1.97	174.76	2.818	O8	[-x+2, -y+1, -z+1]
O11-H11D	0.85	2.005	174.15	2.852	O19	[-x+1, -y, -z+1]
O11-H11E	0.85	1.95	144.76	2.689	O15	
O18-H18C	0.85	1.874	144.2	2.611	O21	
O18-H18D	0.85	1.949	147.61	2.706	O17	
O20-H20B	0.85	1.861	163.1	2.686	O4	[-x+1, -y, -z+1]
O21-H21B	0.85	2.495	125.34	3.066	O5	[x, y, z-1]
N2-H2A	0.86	2.305	166.56	3.148	O4	[-x+1, -y+1, -z+2]
N3-H3B	0.86	2.272	158.04	3.087	O15	[x, y, z+1]
N6-H6A	0.86	2.223	168.12	3.07	O7	[-x+2, -y+1, -z+1]
N7-H7C	0.86	2.349	155.42	3.151	O2	[x+1, y+1, z-1]

Table S3. H-bonding parameters in compound **3**.

**D-H**	**D-H (Å)**	**H…A (Å)**	**<DH…A(º)**	**D…A (Å)**	**A**	**Symmetry**
O7-H7D	0.85	2.253	132.23	2.894	O6	[x, y, z+1]
O7-H7E	0.85	1.895	150.65	2.669	O6	[-x+1, -y+1, -z]
O8-H8D	0.85	1.866	174.59	2.713	O1	[-x+1, -y, -z+1]
O8-H8E	0.85	1.914	148.89	2.678	O4	
O9-H9D	0.85	2.014	155.02	2.807	O3	
O9-H9E	0.85	2.231	138.01	2.919	O5	[-x+1, -y+1, -z]
O10-H10A	0.85	2.348	163.43	3.172	O4	[x+1, y, z]
O10-H10B	0.85	2.256	149.9	3.022	O6	[-x+1, -y+1, -z]
N2-H2A	0.86	1.97	173.08	2.825	O9	[-x+1, -y+1, -z]
N4-H4A	0.86	2.067	178.24	2.927	O10	

Table S4. H-bonding parameters in compound **4**.

**D-H**	**D-H (Å)**	**H…A (Å)**	**<DH…A(º)**	**D…A (Å)**	**A**	**Symmetry**
N2-H2A	0.86	1.921	174.39	2.778	O5	[-x-1/2, -y+1/2, -z]
N3-H3A	0.86	2.14	157.79	2.954	O6	[-x, -y+1, -z]

Table S5. H-bonding parameters in compound **5**.

D-H	D-H (Å)	H…A (Å)	<DH…A(º)	D…A (Å)	A	Symmetry
N2-H2A	0.86	2.159	174.67	3.017	O5	[-x+1, -y+1, -z+1]
N3-H3A	0.86	1.985	179.29	2.845	O1	[x+1, y, z]
O8-H8C	0.826	1.844	155.85	2.619	O5	[x-1, y, z]
O9-H9E	0.774	1.914	174.42	2.686	O3	[-x+1, -y+1, -z+1]
O9-H9F	0.801	1.873	170.1	2.666	O7	[x+1, y-1, z]

Table S6. H-bonding parameters in compound **6**.

D-H	D-H (Å)	H…A (Å)	<DH…A(º)	D…A (Å)	A	Symmetry
O10-H10C	0.85	2.278	113.78	2.734	O12'_b	
O10-H10D	0.85	1.949	138.56	2.647	O7	[-x+2, -y+2, -z+1]
N2-H2A	0.86	2.105	162.63	2.937	O8	[-x+2, -y+1, -z+2]
N3-H3A	0.86	2.11	174.72	2.968	O7	[-x+2, -y+1, -z+2]
N6-H6A	0.86	2.258	169.31	3.107	O1	[x, y+1, z]
N6-H6A	0.86	2.002	177.52	2.862	O1'	[x, y+1, z]
O12_a-H12E_a	0.85	1.685	176.15	2.534	O3	
O12_a-H12F_a	0.85	2.243	178.53	3.093	O5	

Table S7. H-bonding parameters in compound **7**.

D-H	D-H (Å)	H…A (Å)	<DH…A(º)	D…A (Å)	A	Symmetry
N2-H2A	0.86	2.141	166.66	2.985	O7	[x, y+1, z]
N3-H3A	0.86	2.236	152.58	3.025	O6	[-x+3, -y, -z+1]
N5-H5A	0.86	2.308	150.66	3.086	O1	

Table S8. H-bonding parameters in compound **8**.

D-H	D-H (Å)	H…A (Å)	<DH…A(º)	D…A (Å)	A	Symmetry
N2-H2A	0.86	2.176	173.71	3.032	O6	[-x+1, -y+1, -z+1]
N3-H3A	0.86	2.011	171.83	2.865	O7	[x+1/2, y, -z+1/2]
O7-H7C	0.82	2.001	161.17	2.79	O6	

Table S9. H-bonding parameters in compound **9**.

D-H	D-H (Å)	H…A (Å)	<DH…A(º)	D…A (Å)	A	Symmetry
O8-H8B	0.85	2.178	125.33	2.758	O5	[-x+3/2, y-1/2, -z+1/2]
O8-H8C	0.85	1.943	158.65	2.752	O6	
O9-H9C	0.85	2.052	164.95	2.882	O2	
O9-H9D	0.85	1.833	164	2.66	O8	[-x+3/2, y+3/2, -z+1/2]
N2-H2A	0.86	2.488	127.66	3.089	O7	[x, y+1, z]
N3-H3A	0.86	2.134	156.58	2.943	O9	[-x+1, -y+2, -z]
N5-H5A	0.86	2.515	129.03	3.129	O9	[x, -y+1, z+1/2]

Table S10. H-bonding parameters in compound **10**.

D-H	D-H (Å)	H…A (Å)	<DH…A(°)	D…A (Å)	A	Symmetry
N2-H2A	0.86	2.118	149.28	2.891	O7	[x, -y+3/2, z-1/2]
N3-H3A	0.86	2.007	178.03	2.867	O4	[-x, y+1/2, -z+3/2]
N5-H5A	0.86	2.062	169.63	2.912	O9	
O8-H8C	0.85	2.177	148.29	2.934	O1	
O8-H8D	0.85	2.479	116.75	2.961	O2	[-x, y-1/2, -z+3/2]
O9-H9C	0.85	2.307	170.62	3.148	O4	[-x+1, -y+1, -z+2]

Table S11. The percentage of MB (pmb) remaining in solution and degradation efficiency (de) with the mean values and standard deviations for catalytic experiments for complex **9** and **10**.

Tube 1: MB solution

	1st	2nd	3rd	Mean	Standard Deviation
0	100	100	100	100	0
15	97.06	94.40	93.89	95.12	1.70
30	95.40	90.39	89.72	91.84	3.10
45	93.38	86.69	87.17	89.08	3.73
60	92.46	82.90	83.77	86.38	5.29
de	7.540	17.10	16.23	13.62	5.29

Tube 2: MB + H_2O_2

	1st	2nd	3rd	Mean	Standard Deviation
0	100	100	100	100	0
15	72.64	77.95	76.24	75.61	2.71
30	52.15	53.66	51.16	52.32	1.26
45	36.05	37.37	35.60	36.34	0.92
60	26.53	27.13	25.54	26.40	0.80
de	73.47	72.87	74.46	73.60	0.80

Tube 3 for **9**: MB + 9

	Complex **9**				
	1st	2nd	3rd	Mean	Standard Deviation
0	100	100	100	100	0
15	95.59	93.41	92.31	93.77	1.67
30	92.52	89.02	86.41	89.32	3.06
45	89.65	84.71	82.93	85.76	3.48
60	88.40	80.47	78.64	82.50	5.19
de	11.60	19.53	21.36	17.50	5.19

Table S11 continue. The percentage of MB (pmb) remaining in solution and degradation efficiency (de) with the mean values and standard deviations for catalytic experiments for complex **9** and **10**. (cont.)

Tube 4 for **9**: MB + **9** + H_2O_2

	Complex **9**				
	1st	2nd	3rd	Mean	Standard Deviation
0	100	100	100	100	0
15	66.19	68.59	65.46	66.75	1.64
30	41.60	41.77	41.16	41.51	0.31
45	27.76	26.42	25.75	26.64	1.02
60	19.60	18.20	17.51	18.44	1.06
de	80.40	81.80	82.49	81.56	1.06

Tube 3 for **10**: MB + **10**

	Complex **10**				
	1st	2nd	3rd	Mean	Standard Deviation
0	100	100	100	100	0
15	90.28	89.43	80.58	86.76	5.37
30	83.10	84.19	86.14	84.48	1.54
45	77.55	80.06	82.71	80.11	2.58
60	73.53	77.64	80.60	77.26	3.55
de	26.47	22.36	19.40	22.74	3.55

Tube 4 for **10**: MB + **10** + H_2O_2

	Complex **10**				
	1st	2nd	3rd	Mean	Standard Deviation
0	100	100	100	100	0
15	56.05	50.67	53.93	53.55	2.71
30	35.10	28.70	29.78	31.19	3.43
45	24.19	18.24	18.99	20.47	3.24
60	17.40	12.85	13.37	14.54	2.49
de	82.60	87.15	86.63	85.46	2.49

Figure S1. Chain arrangement in **1**. (a) Hydrogen bonding utilized to extend three-dimensional structure. (b) Ball and stick model view in chain arrangement.

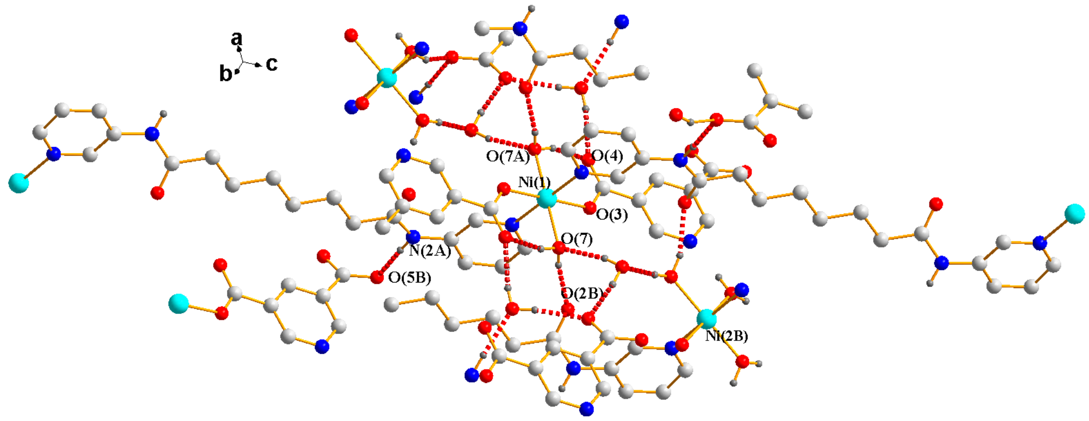

(a)

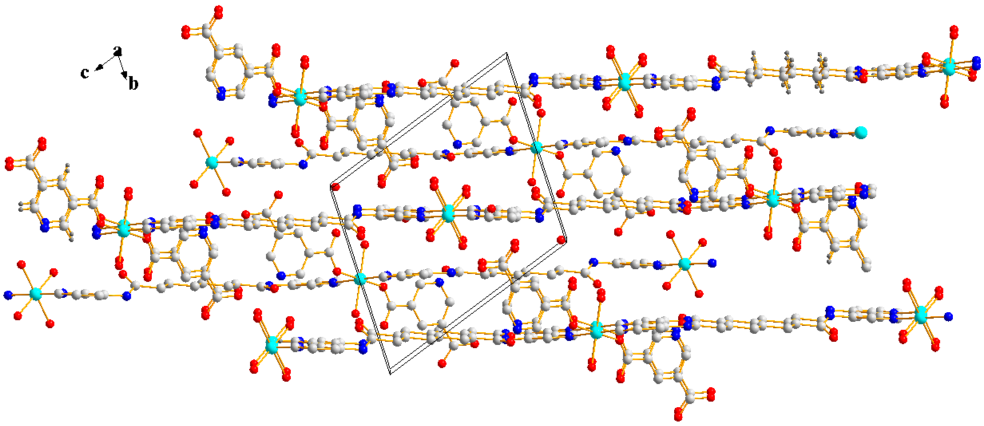

(b)

Figure S2. Layer arrangement in **2**. (a) Ball and stick model view. (b) Topological view.

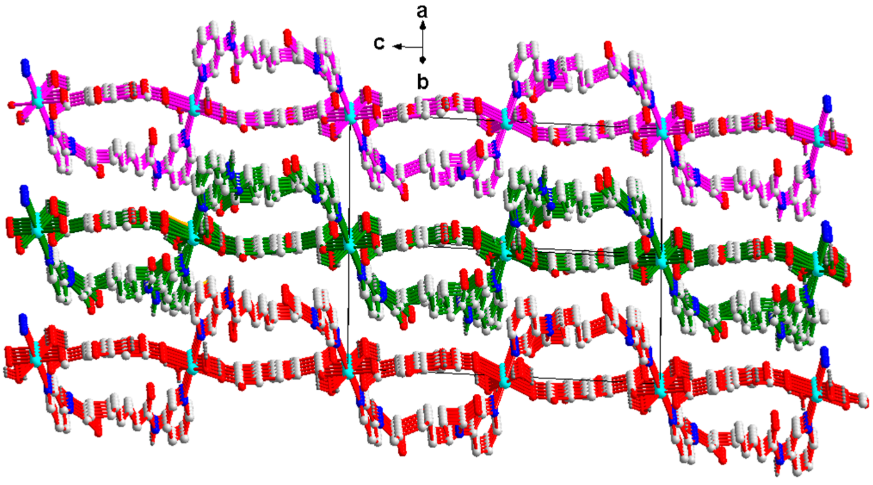

(a)

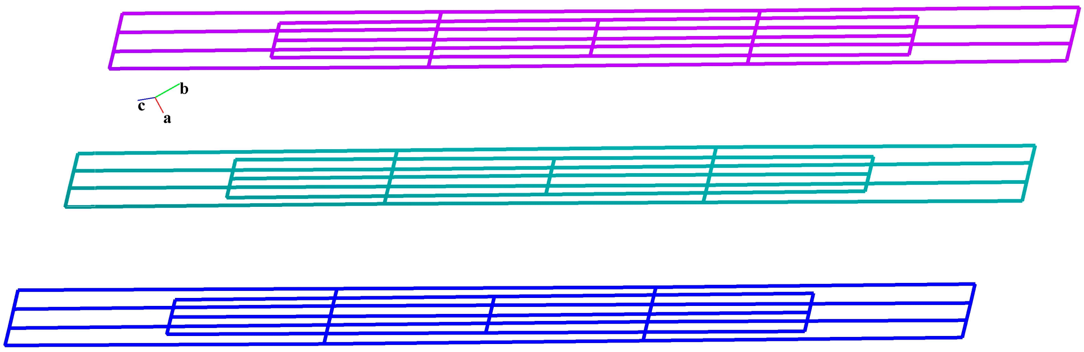

(b)

Figure S3. Layer arrangement in **3**. (a) Ball and stick model view. (b) Topological view.

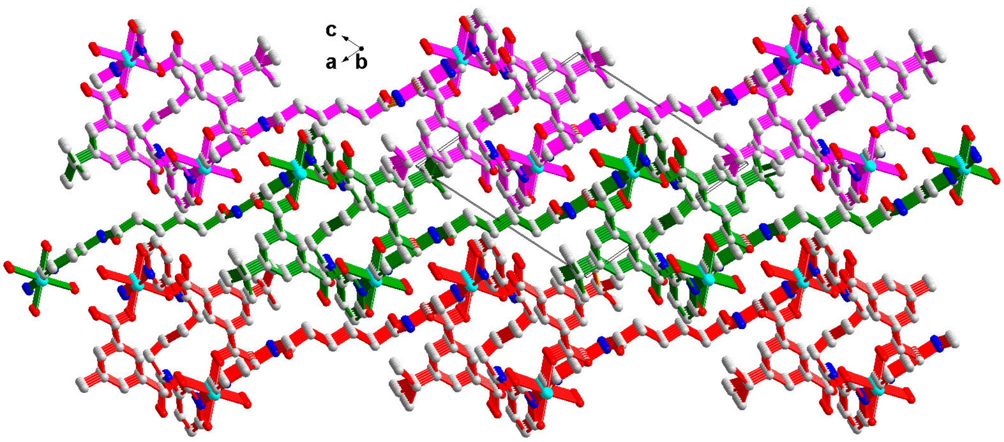

(a)

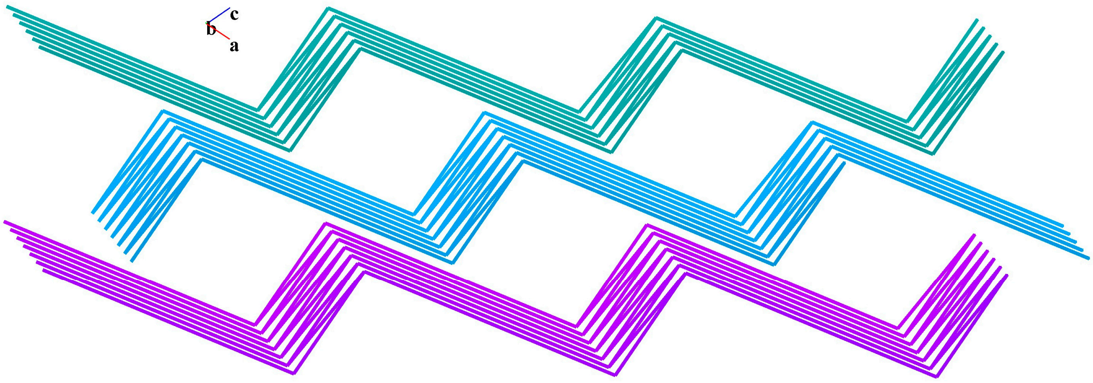

(b)

Figure S4. Layer arrangement in **5**. (a) Ball and stick model view. (b) Topological view.

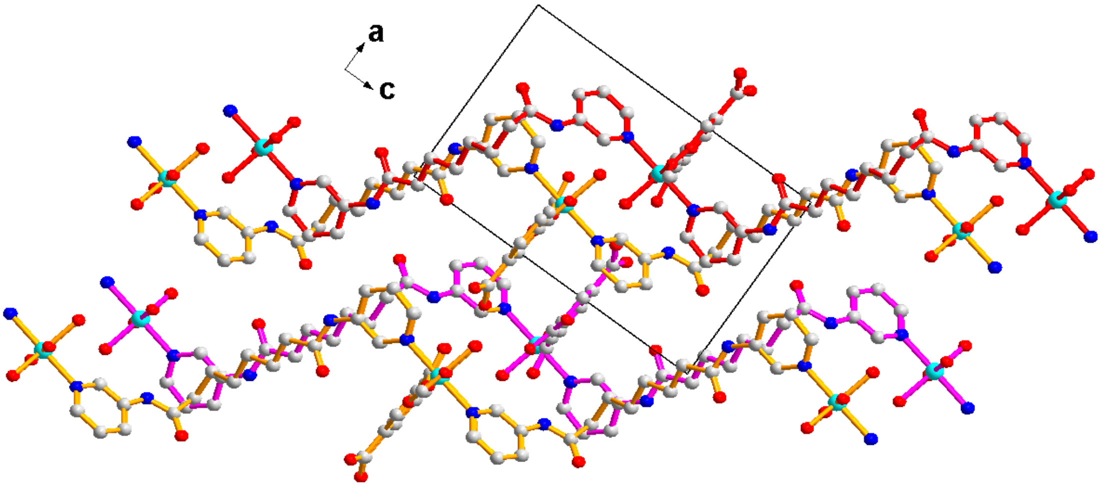

(a)

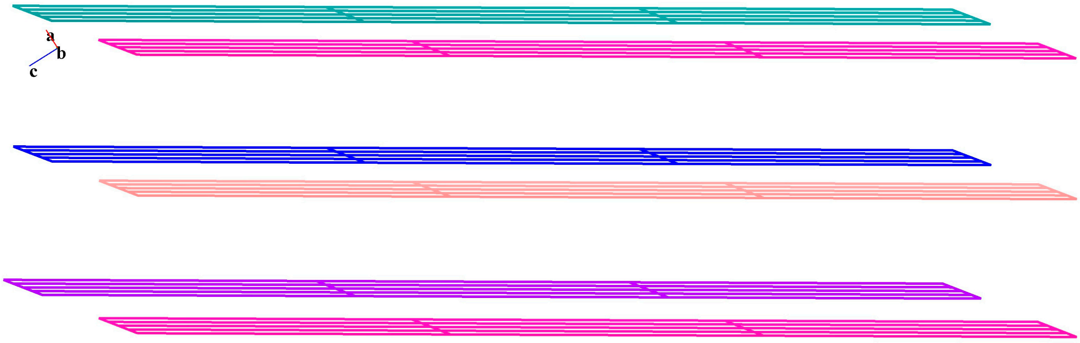

(b)

Figure S5. Layer arrangement in **6**. (a) Ball and stick model view. (b) Topological view.

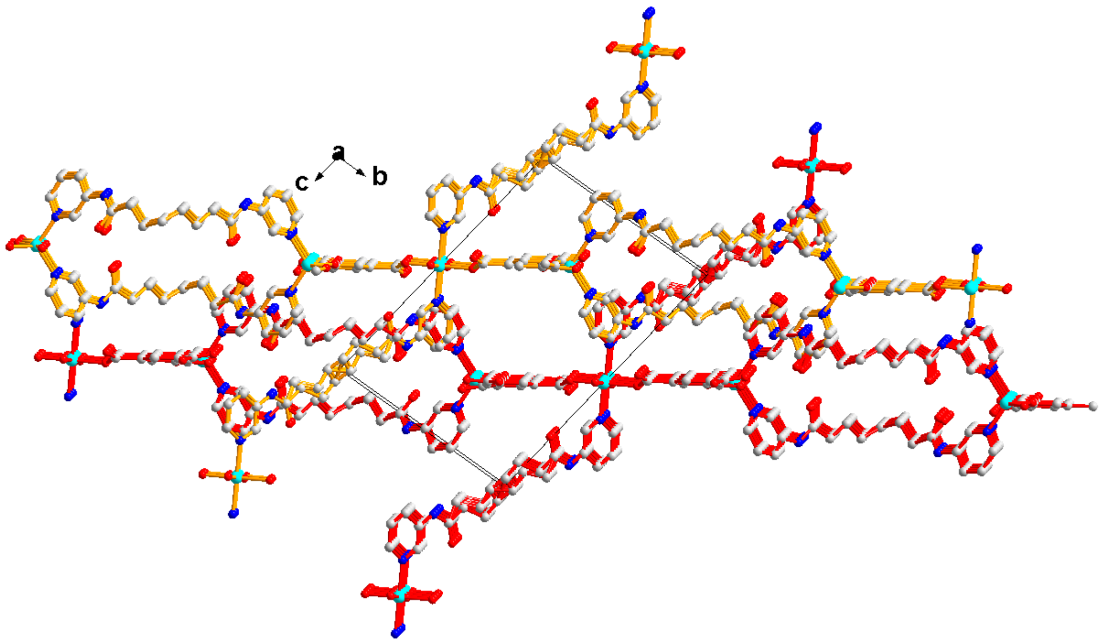

(a)

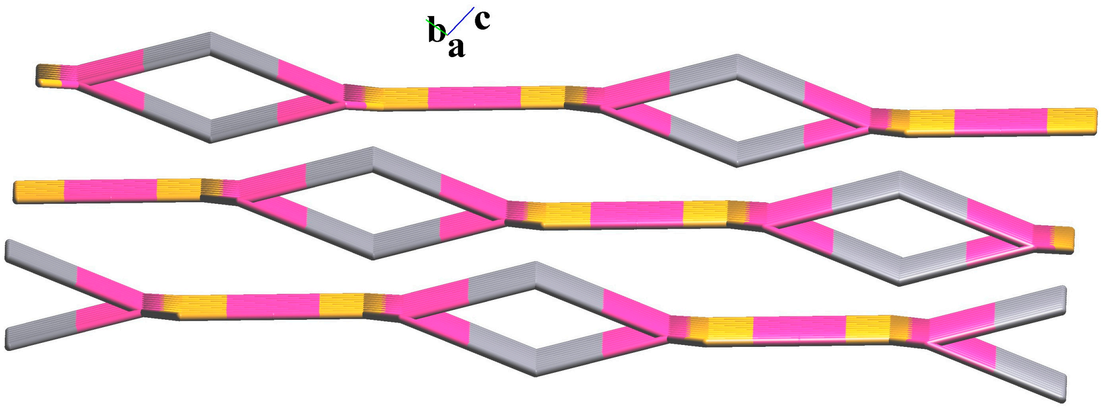

(b)

Figure S6. Layer arrangement in **7**. (a) Ball and stick model view. (b) Topological view.

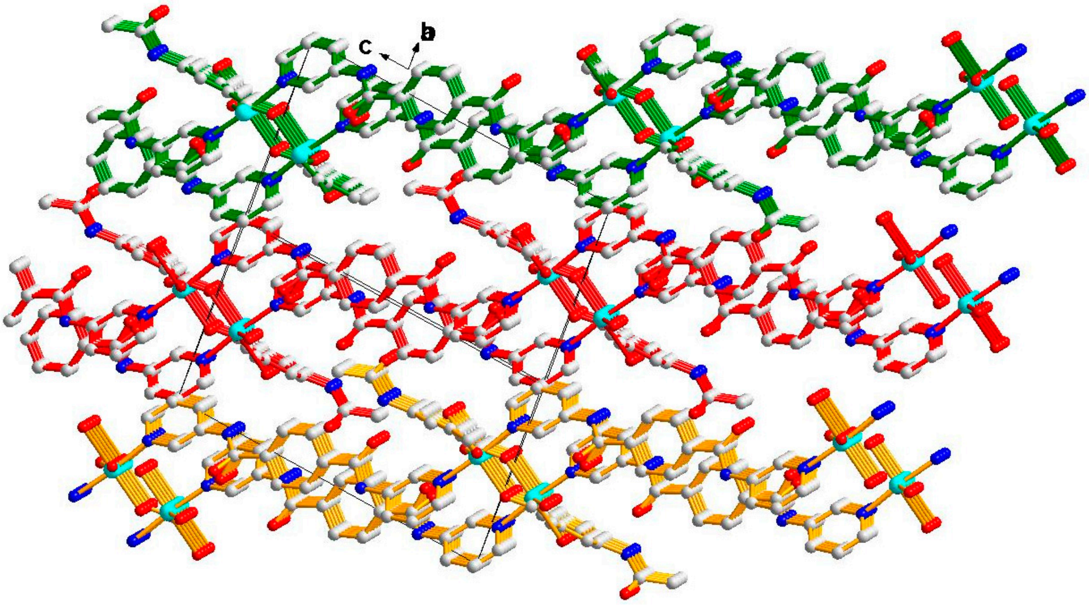

(a)

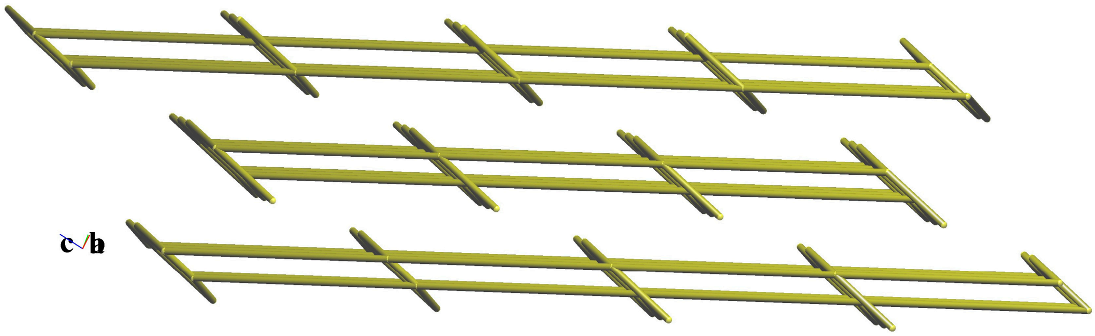

(b)

Figure S7. The excitation and emission spectra of free ligand of $\mathbf{L^2}$.

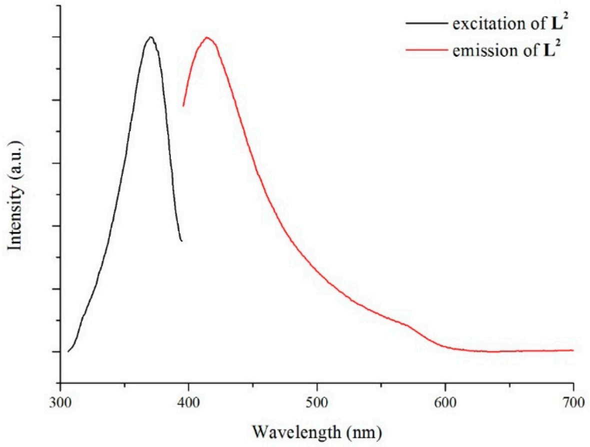

Figure S8. The excitation and emission spectra of free ligand of $\mathbf{L^4}$.

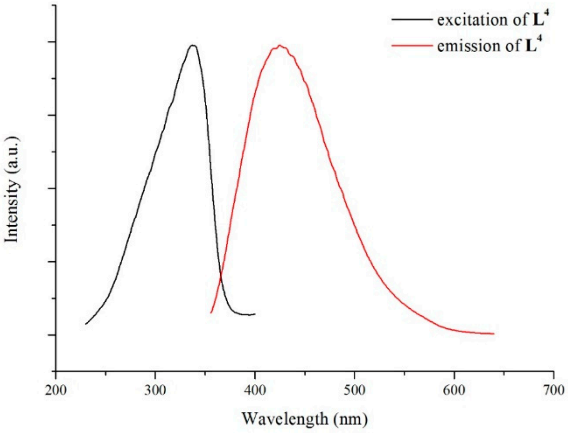

Figure S9. The excitation and emission spectra of free ligand of H_2AIPA.

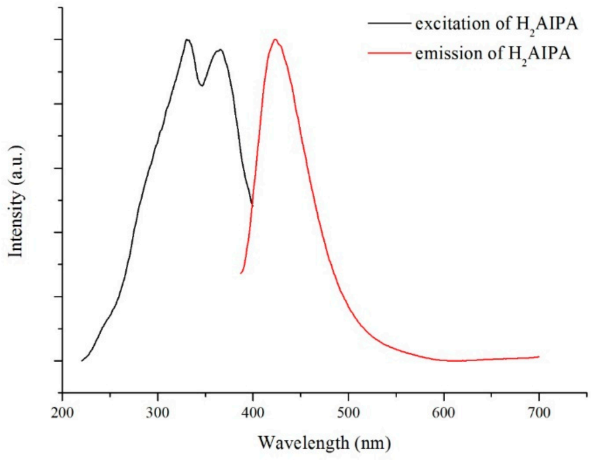

Figure S10. The excitation and emission spectra of complex **9**.

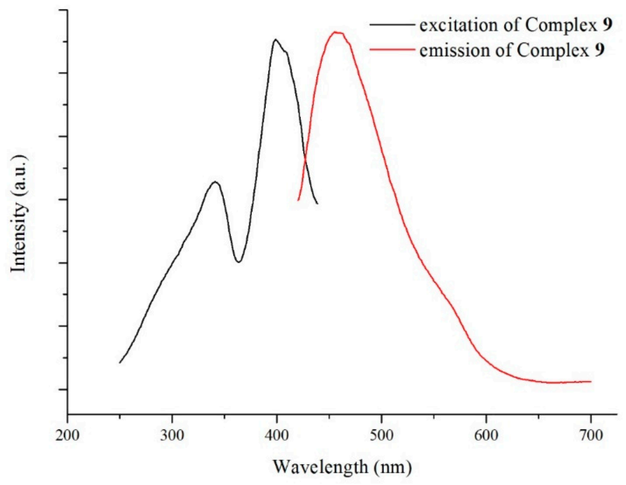

Figure S11. The excitation and emission spectra of complex **10**.

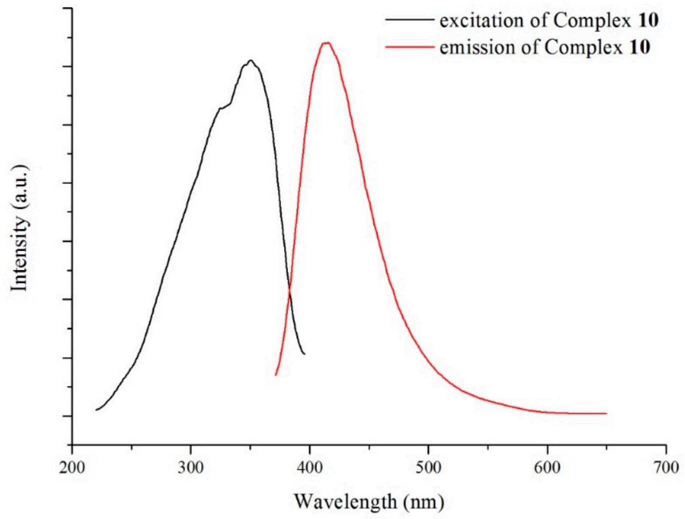

Figure S12. The UV-vis spectra of tube 1-experiment 1

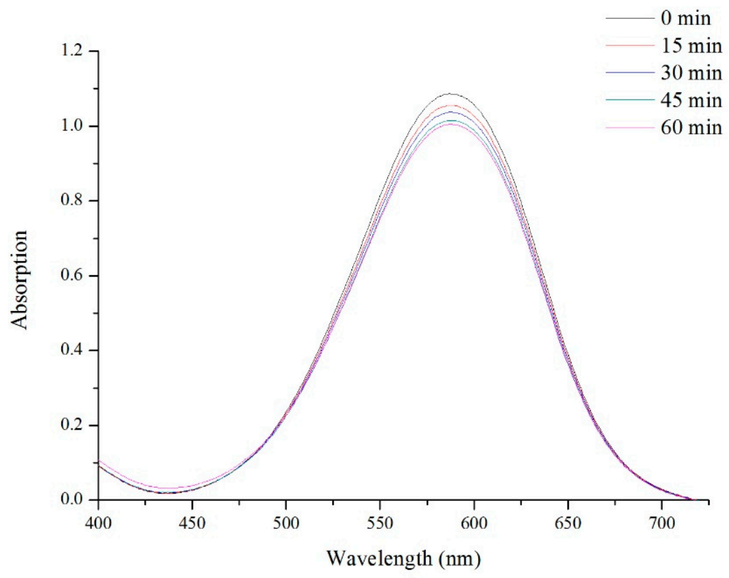

Figure S13. The UV-vis spectra of tube 2-experiment 1.

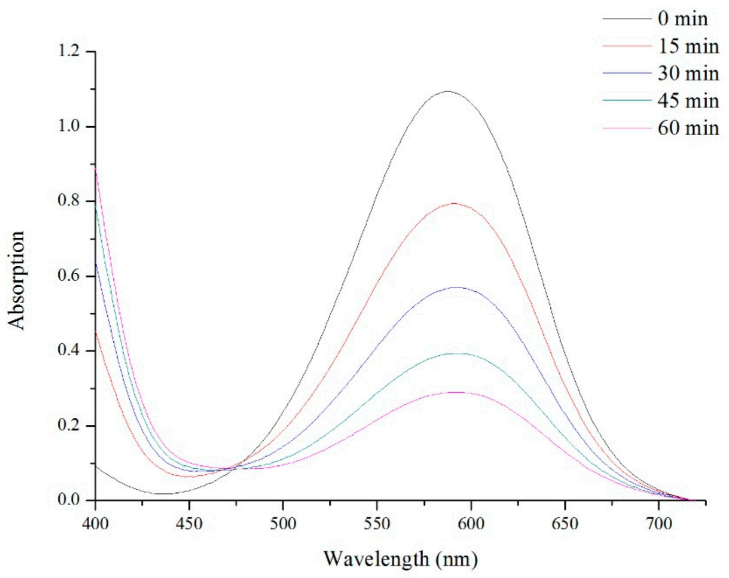

Figure S14. The UV-vis spectra of tube 3-experiment 1 for complex **9**.

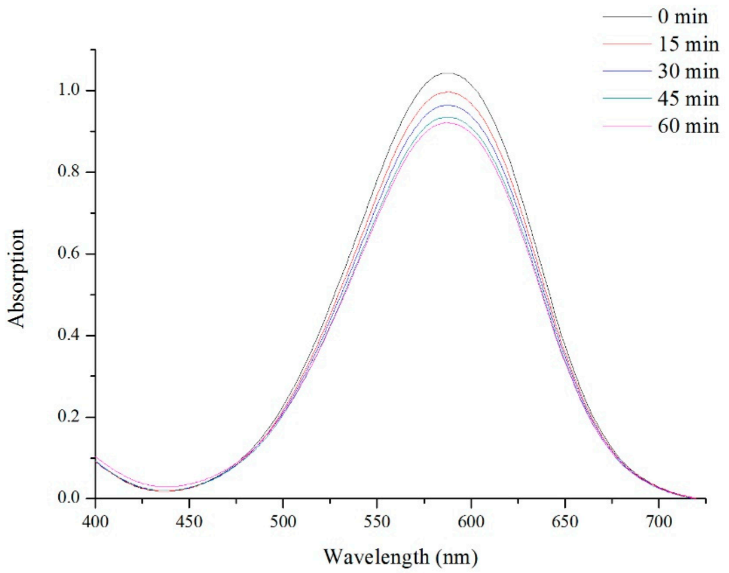

Figure S15. The UV-vis spectra of tube 4-experiment 1 for complex **9**.

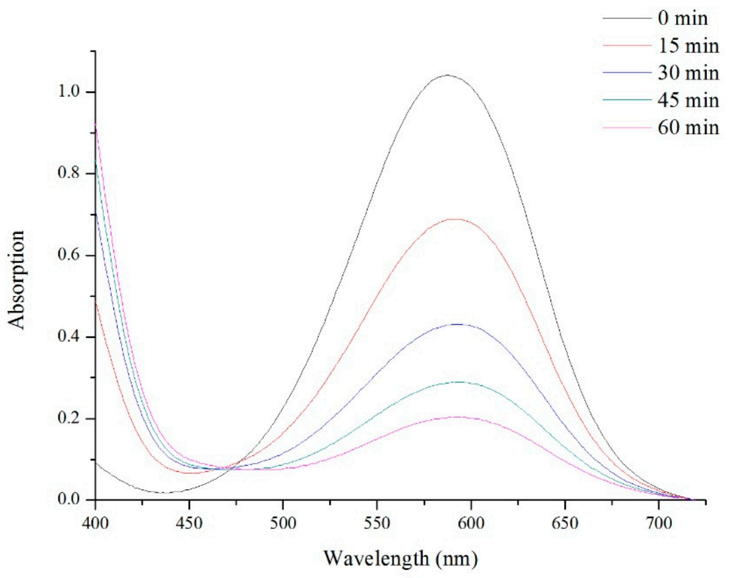

Figure S16. The UV-vis spectra of tube 1-experiment 2.

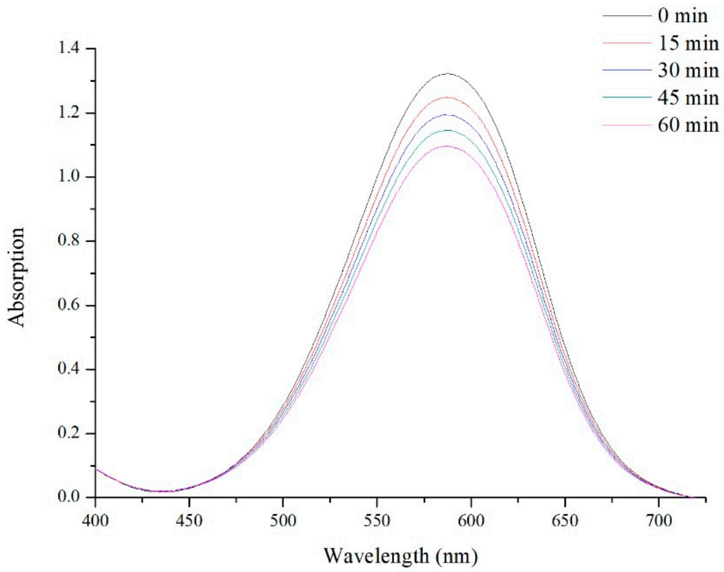

Figure S17. The UV-vis spectra of tube 2-experiment 2.

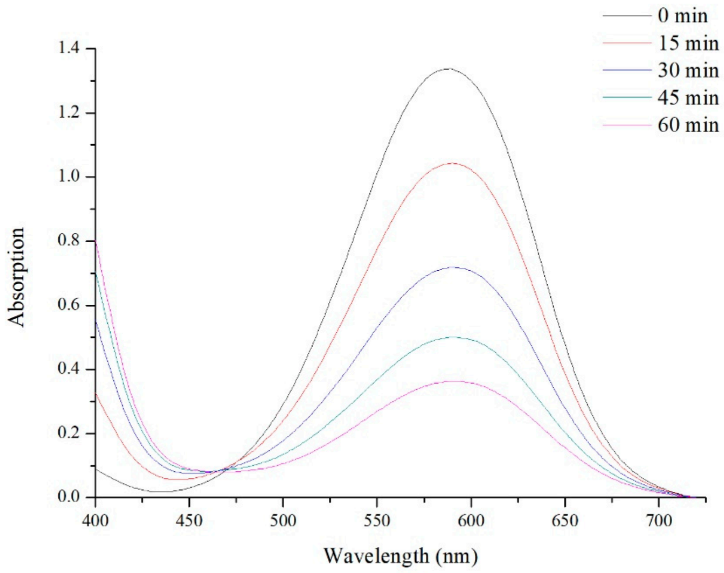

Figure S18. The UV-vis spectra of tube 3-experiment 2 for complex **9**.

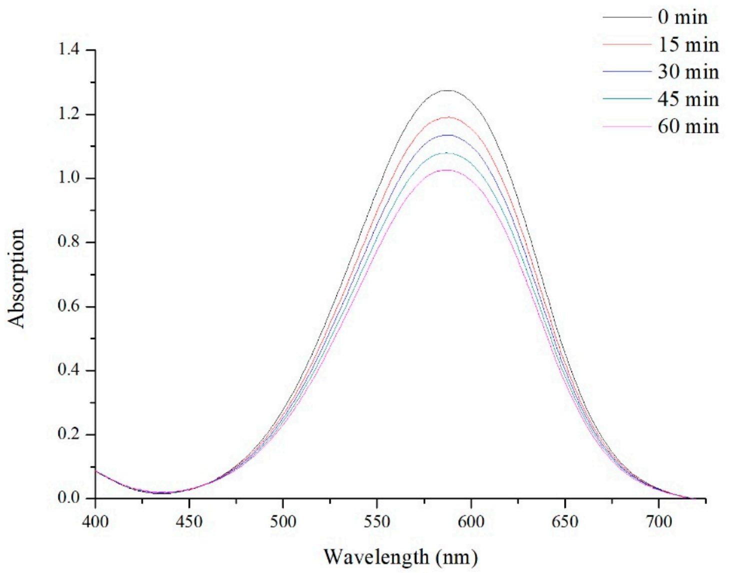

Figure S19. The UV-vis spectra of tube 4-experiment 2 for complex **9**.

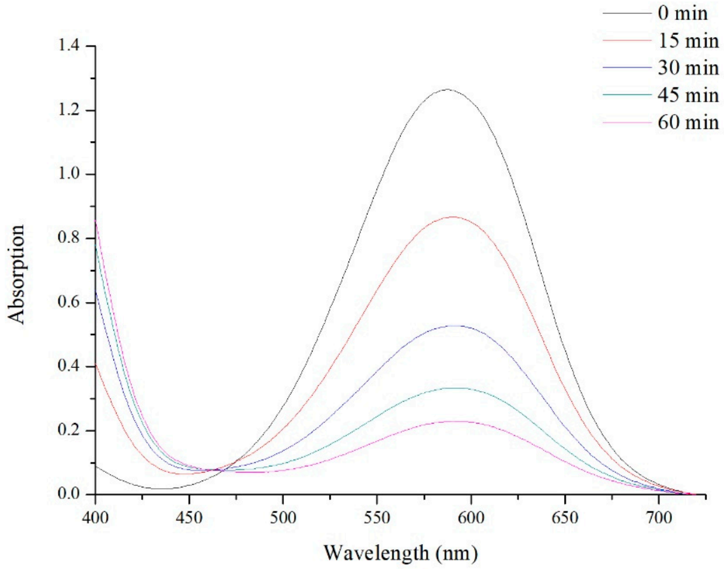

Figure S20. The UV-vis spectra of tube 1-experiment 3.

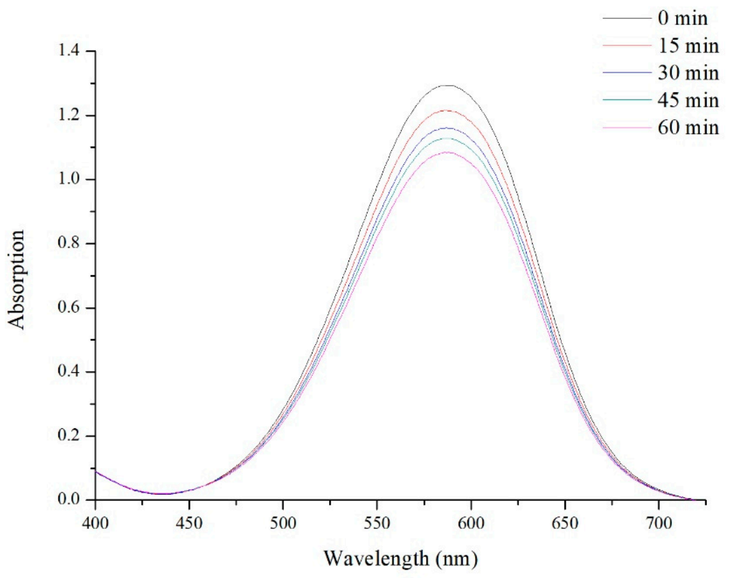

Figure S21. The UV-vis spectra of tube 2-experiment 3.

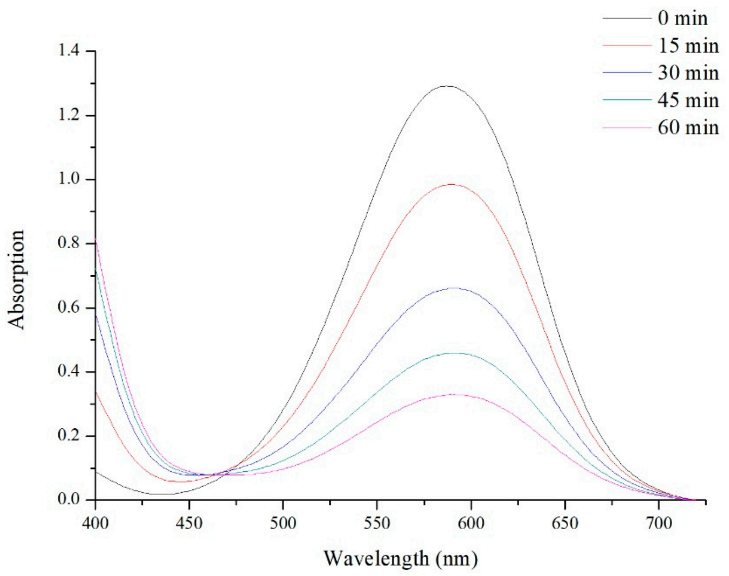

Figure S22. The UV-vis spectra of tube 3-experiment 3 for complex **9**.

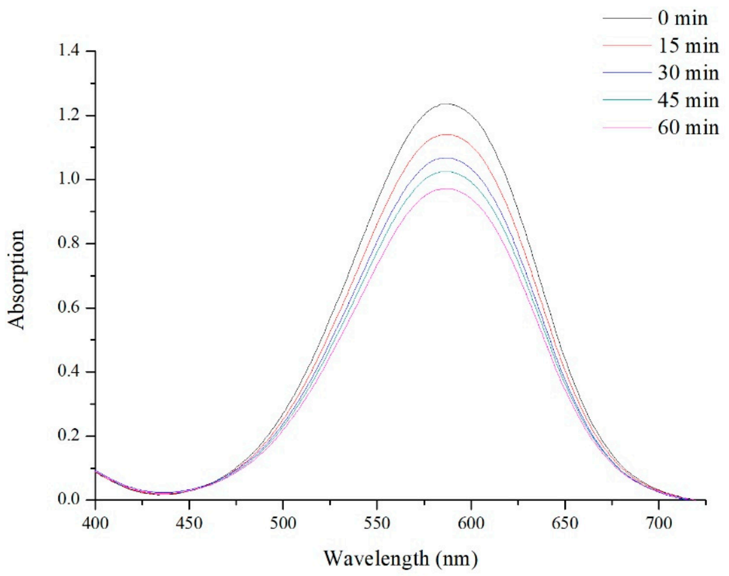

Figure S23. The UV-vis spectra of tube 4-experiment 3 for complex **9**.

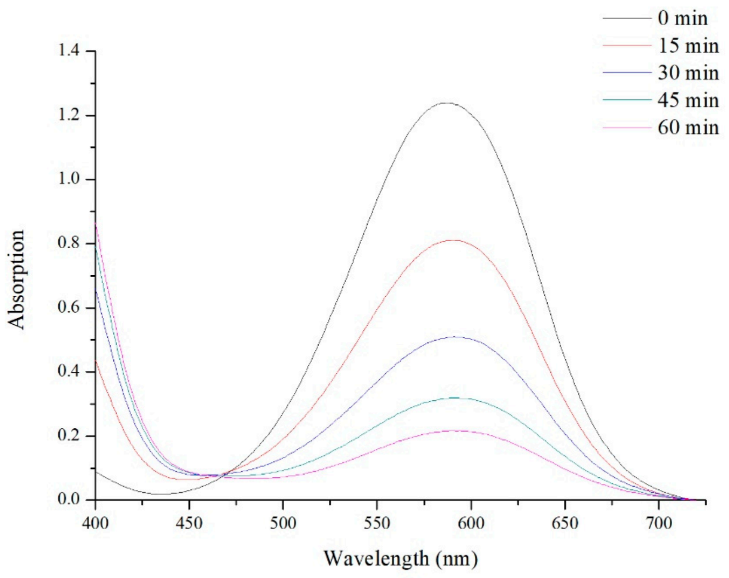

Figure S24. The UV-vis spectra of tube 3-experiment 1 for complex **10**.

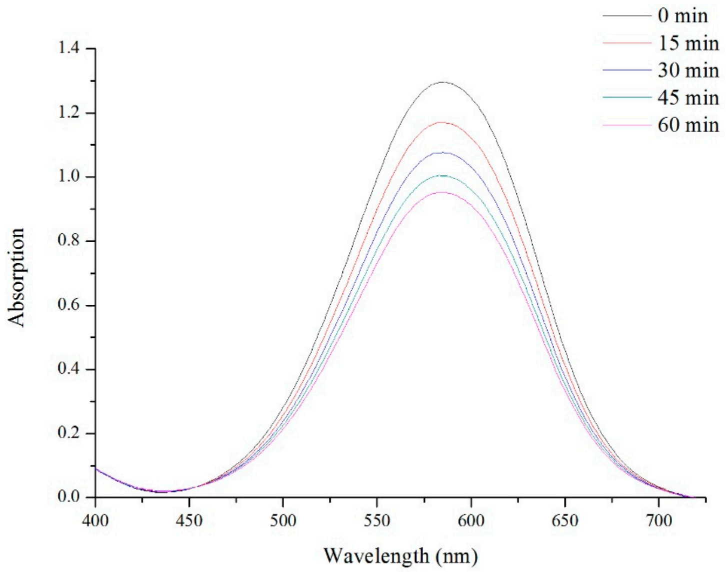

Figure S25. The UV-vis spectra of tube 4-experiment 1 for complex **10**.

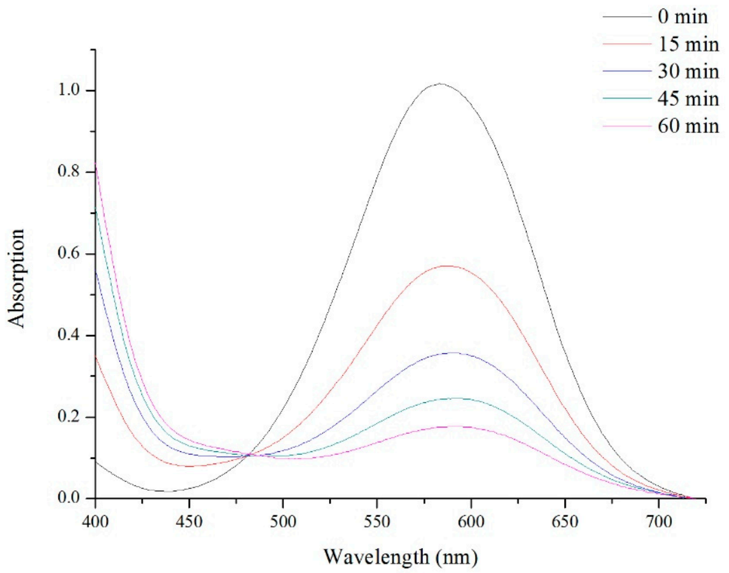

Figure S26. The UV-vis spectra of tube 3-experiment 2 for complex **10**.

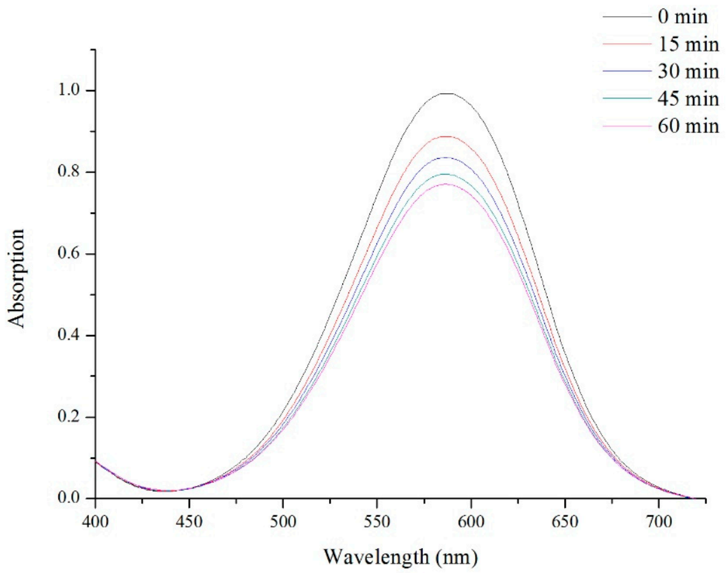

Figure S27. The UV-vis spectra of tube 4-experiment 2 for complex **10**.

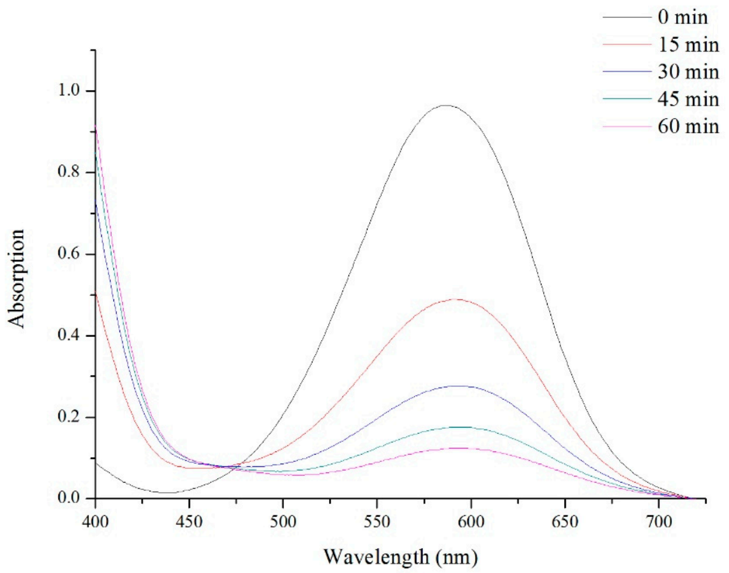

Figure S28. The UV-vis spectra of tube 3-experiment 3 for complex **10**.

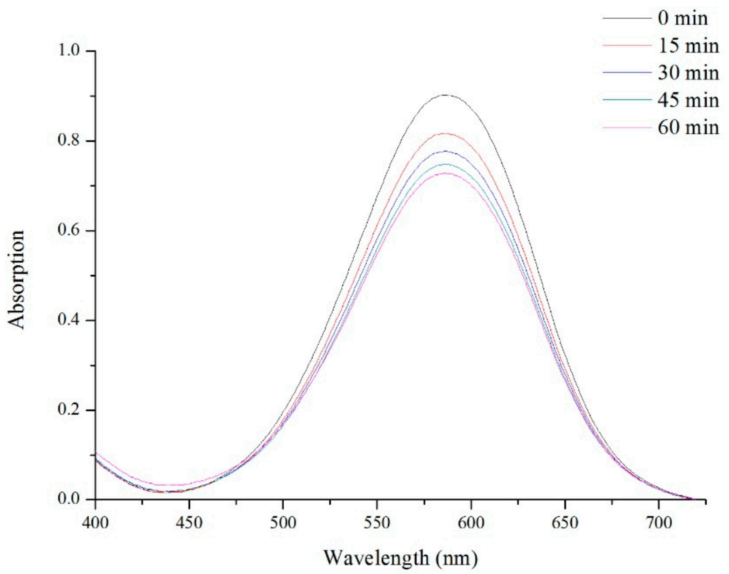

Figure S29. The UV-vis spectra of tube 4-experiment 3 for complex **10**.

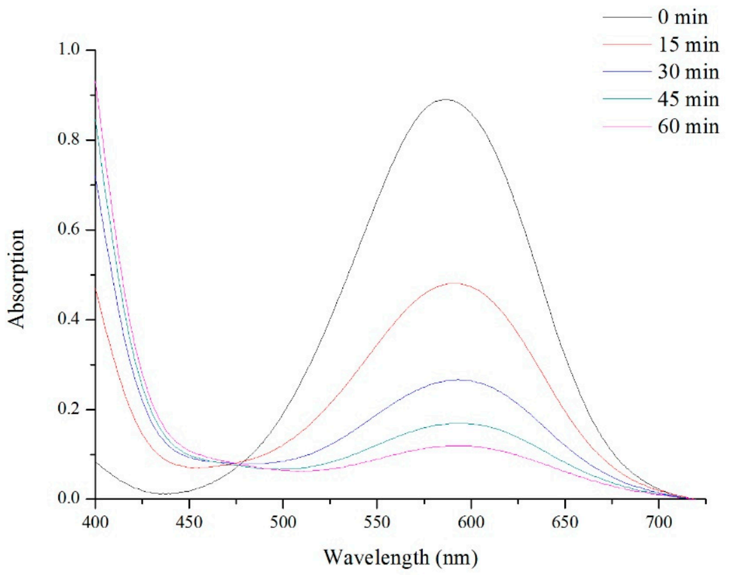

Figure S30. The PXRD patterns of complexes **9** and **10** and after photocatalytic degradation of MB.

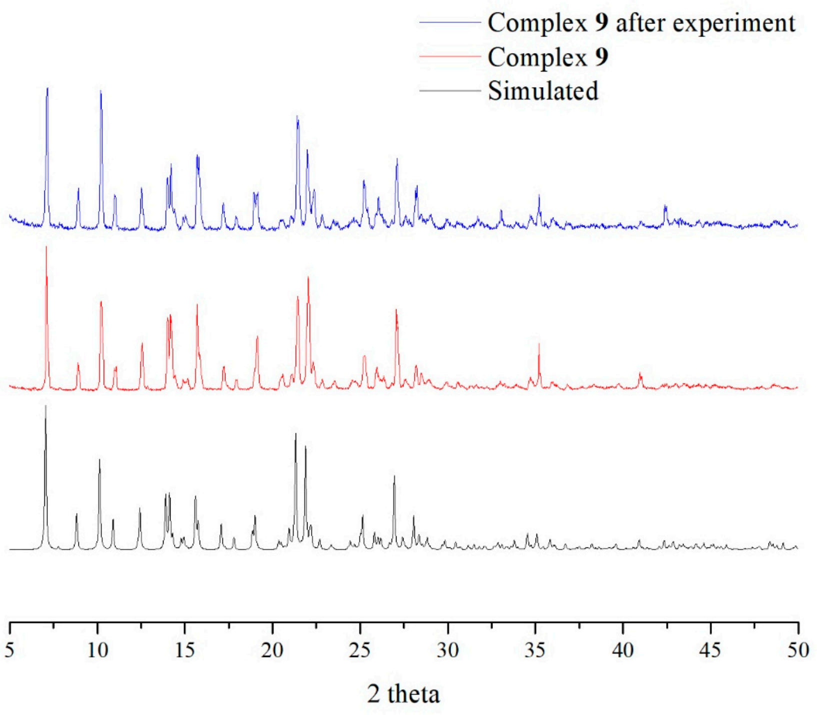

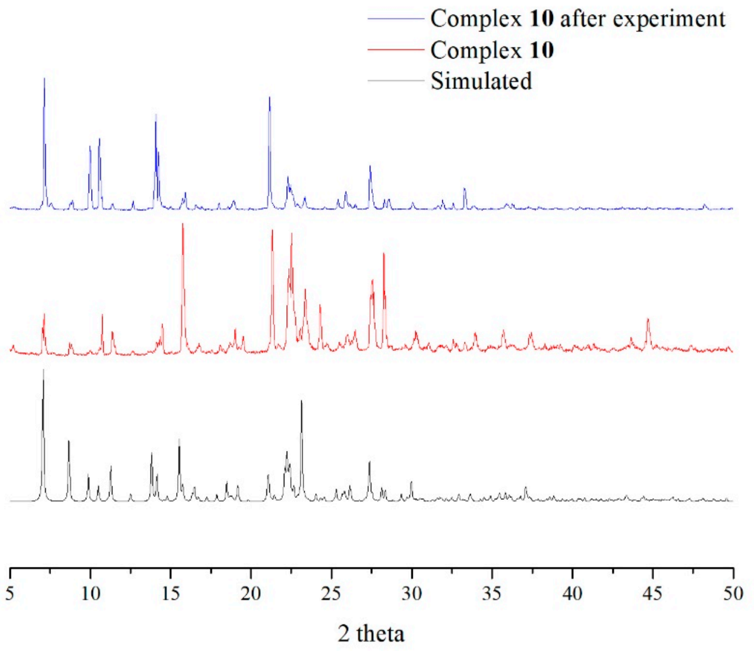

Figure S31. The PXRD patterns for complex **1**.

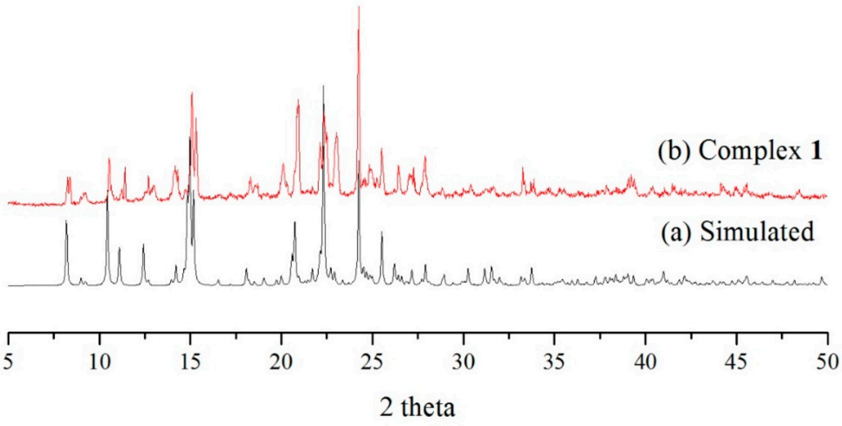

Figure S32. The PXRD patterns for complex **2**.

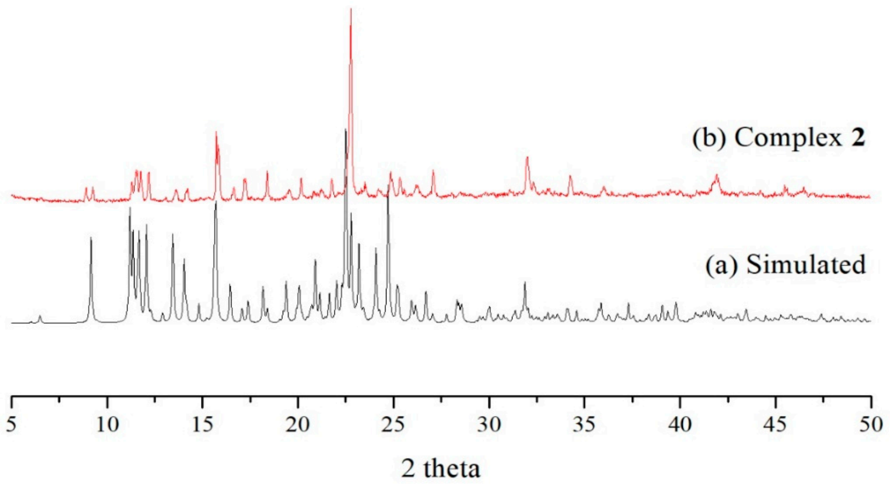

Figure S33. The PXRD patterns for complex **3**.

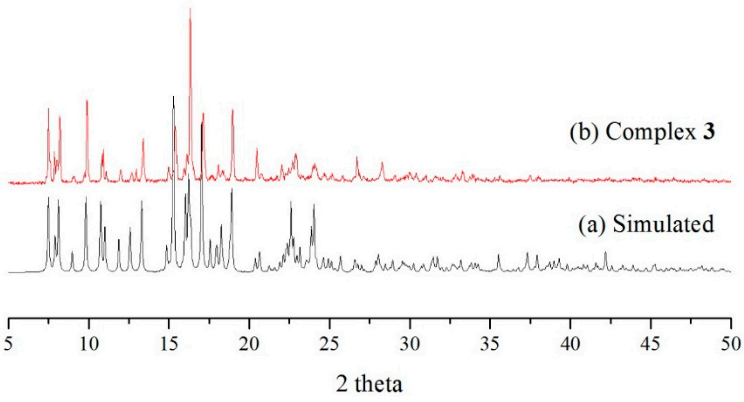

Figure S34. The PXRD patterns for complex **4**.

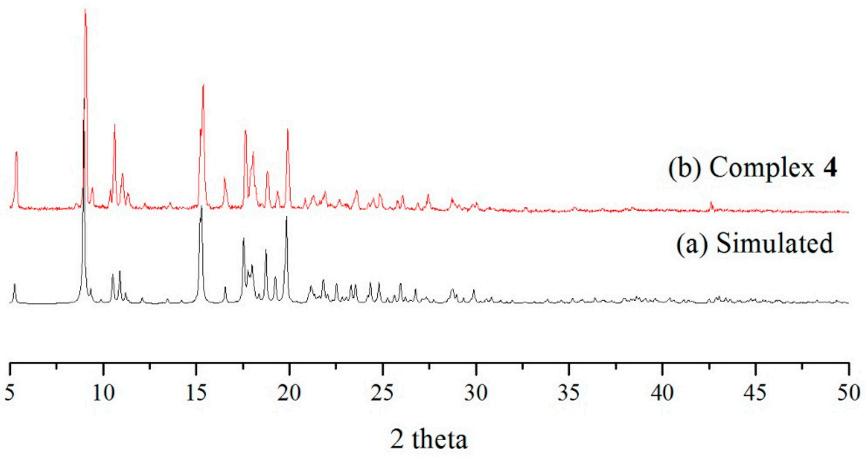

Figure S35. The PXRD patterns for complex **5**.

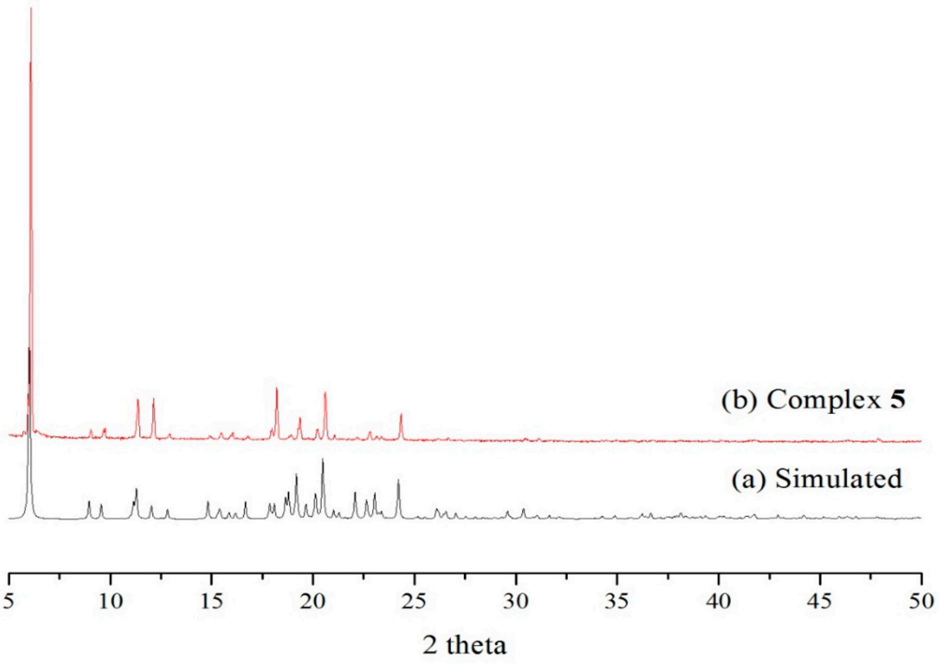

Figure S36. The PXRD patterns for complex **6**.

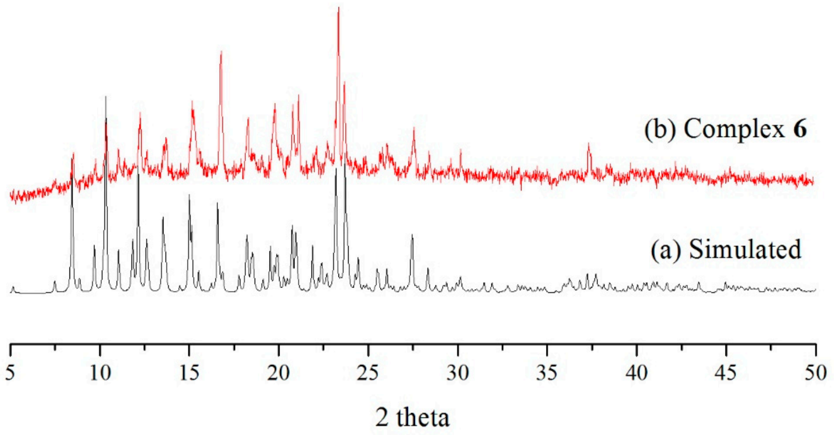

Figure S37. The PXRD patterns for complex **7**.

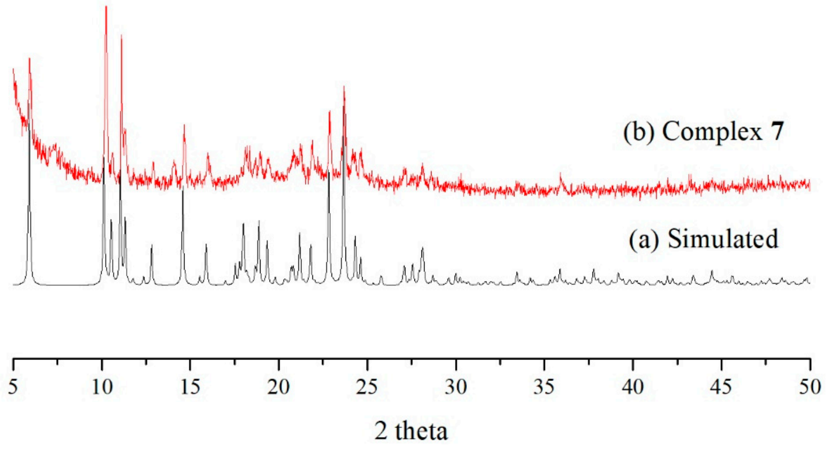

Figure S38. The PXRD patterns for complex **8**.

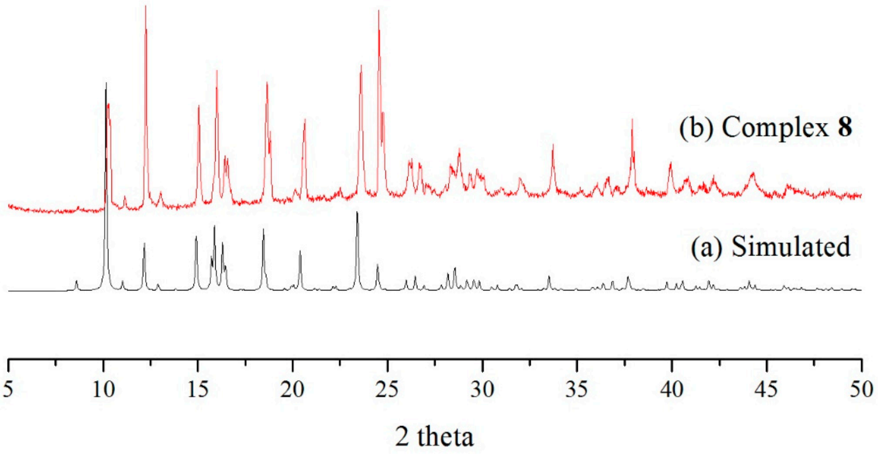

Figure S39. The PXRD patterns for complex **9**.

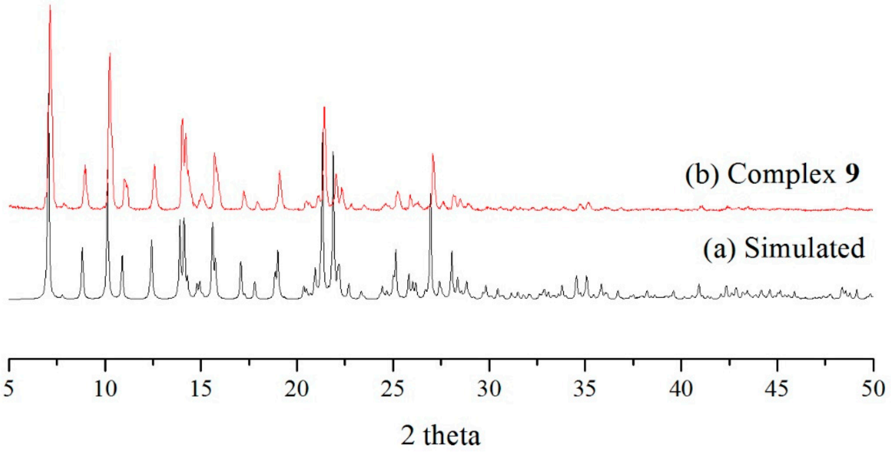

Figure S40. The PXRD patterns for complex **10**.

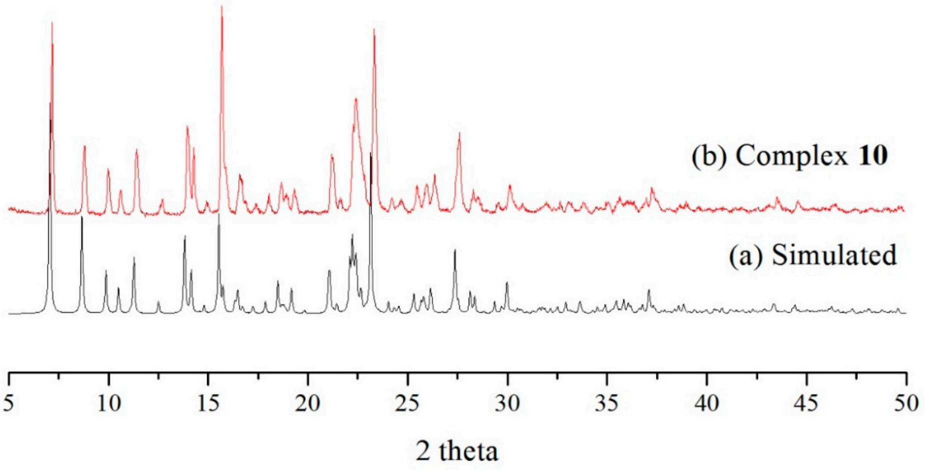

Scheme 1. Chemical structure of Methyl Blue.

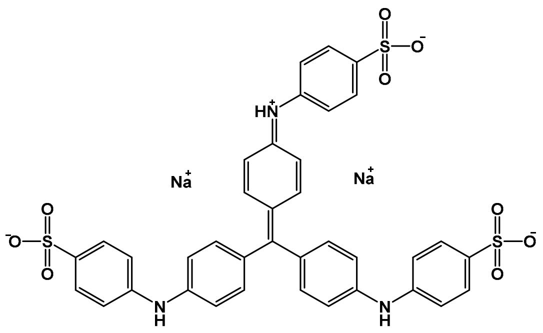

Supplement: Supplementary file 1 [file polymers-09-00691-s001.pdf]
